# Temporality modulates the effect of network heterogeneity on cooperation fixation

**DOI:** 10.1038/s41467-026-72717-w

**Published:** 2026-05-08

**Authors:** Aming Li, Yao Meng, Lei Zhou, Naoki Masuda, Long Wang

**Affiliations:** 1 Center for Systems and Control, School of Advanced Manufacturing and Robotics, Peking University, Beijing, China; 2 Research Center for Robotics, Peking University, Beijing, China; 3 Center for Multi-Agent Research, Institute for Artificial Intelligence, Peking University, Beijing, 100871 China; 4 School of Future Technology, Shandong University, Jinan, China; 5 School of Automation, Beijing Institute of Technology, Beijing, China; 6 Gilbert S. Omenn Department of Computational Medicine and Bioinformatics, University of Michigan, Ann Arbor, MI USA; 7 Department of Mathematics, University of Michigan, Ann Arbor, MI USA; 8 Department of Mathematics, State University of New York at Buffalo, BUF New York, USA; 9 Computational and Data-Enabled Science and Engineering Program, State University of New York at Buffalo, BUF New York, USA

**Keywords:** Social evolution, Complex networks

## Abstract

Understanding the evolution of cooperation in structured populations remains a central challenge in multidisciplinary areas. Although previous findings suggest that structural heterogeneity in static networks hinders cooperation, real-world interactions in most natural and social systems are dynamic and best represented as temporal networks. Here, we challenge this conventional wisdom and, by developing a systematic mathematical framework, we report that structural heterogeneity in temporal networks can instead promote collective cooperation. Importantly, we reveal that such advantages depend on an often-overlooked metric—fixation time—quantifying the time required for a single cooperator to drive the entire population to cooperation. Highly heterogeneous networks accelerate this process within each subnetwork, resulting in a quantitative enhancement of cooperation in temporal networks compared to their homogeneous counterparts. By validating our results on empirical datasets through theoretical analyses and simulations, we provide a consistent framework for analysing cooperative dynamics across static and temporal networked systems.

## Abstract (web summary)

Previous work suggests that heterogeneous static networks hinder collective cooperation fixation. Here, the authors show that heterogeneous temporal networks outperform homogeneous ones by accelerating cooperative spread—an effect washed out in static cases.

## Introduction

The emergence of cooperation through strategy competition and replacement in intelligent systems of interacting individuals is a crucial phenomenon^1–10^. Patterns of interaction among individuals can often be modelled by networks, where nodes indicate individuals, and links represent who interacts with whom^11–14^. Even before the empirical understanding of how humans or animals are connected as networks, Nowak and May showed that homogeneous spatial structures promote cooperation under the prisoner’s dilemma^5^. These and many other studies^11,15–19^ evaluate the equilibrium frequency of cooperators starting from an equal number of cooperators and defectors. Beyond this, a drastically different framework evaluates the evolutionary success of cooperation with fixation probability, which captures the ability of one cooperator to take over the entire population^12,20–22^. A well-known result reported that cooperation is favoured for evolutionary success when the benefit provided by the cooperator over the cost paid exceeds the number of neighbours in homogeneous networks^12^.

In fact, many real-world complex networks exhibit heterogeneous degree distributions, with scale-free networks being a typical example in which the degree obeys a power-law distribution^23,24^. Although heterogeneous networks are reported to increase the equilibrium frequency of cooperators universally compared to the homogeneous counterparts^11,25,26^, the heterogeneity is shown to hinder the fixation of cooperation relative to random regular networks^12,21,27^. This raises an often unstated fact in the evolution of cooperation on prevailing degree-heterogeneous networks—the equilibrium and fixation probability analyses lead to incongruent conclusions under different update rules^10^. Since experimental studies^28^ to validate the results on the evolution of cooperation on complex networks are relatively sparse in general, phenomena that are consistent across different theoretical frameworks are there to be discovered.

So far, numerous accumulating data have verified that many empirical networks are rather time-varying, with the aggregated static networks being heterogeneous^15,29,30^. A key feature of such temporal networks is that interactions are not continuously present but instead appear and disappear over time, leading to intermittent links that static structures cannot capture^29,31^. Such temporality has been shown to strongly influence dynamical processes such as epidemic spreading, information diffusion, and synchronization^31,32^. In evolutionary game theory, temporal structures introduce an additional dimension of heterogeneity: not only who interacts with whom matters, but also when interactions occur. Recent studies suggest that the duration of temporal interactions can fundamentally reshape cooperative dynamics beyond what is observed on time-aggregated static networks^15,33^. Therefore, understanding the role of temporal networks in the evolution of cooperation is crucial for bridging theoretical predictions with realistic, time-varying social and biological systems.

On the other hand, in many natural and human social structures, the evolutionary process that occurs among individuals may be faster than the structural changes in which they are embedded^34,35^. For instance, the structure of ecological communities may change on a seasonal or even annual time scale, yet some species are highly likely to compete and reproduce for multiple generations in a single season^34,36^. In human societies, individuals’ decision-making may evolve more frequently than their social relationships change. Analogous to the scenario in static networks, the exogenous temporality within heterogeneous networks has been validated to enhance the equilibrium frequency of cooperators more effectively than homogeneous counterparts^15^. However, when it comes to the fixation of cooperation, the underlying mechanisms remain rather elusive.

**Fig. 1:**
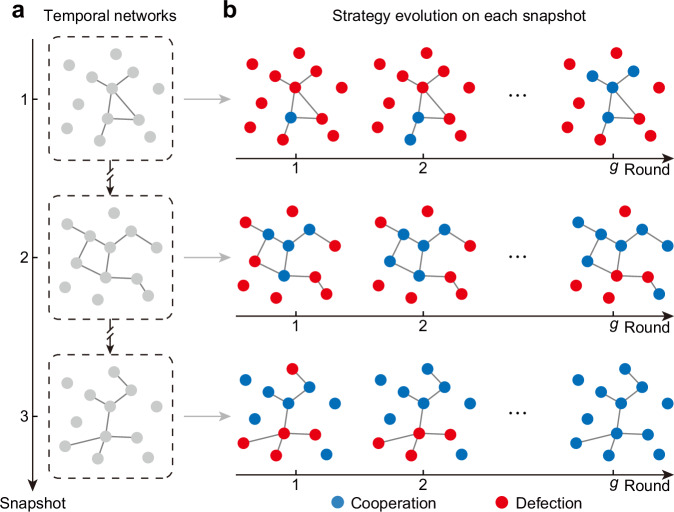
Illustration of strategy evolution on temporal networks. **a** We illustrate the cooperative dynamics on temporal networks containing three snapshots, with structures in each snapshot varying arbitrarily. **b** Individual strategies evolve in finite rounds of games and strategy updates on each snapshot. In each round, individuals choose either cooperation (blue nodes) or defection (red nodes), and play games with their neighbours connected by network links. Afterwards, a random individual is chosen to update its strategy by imitating its neighbours or keeping its own strategy. The snapshot switches to the next subnetwork every *g* rounds of games and updates, and the evolutionary process on temporal networks ends when systems reach full cooperation or defection.

Here we investigate the fixation of cooperation on temporal networks through analysing the cooperative dynamics over subnetworks (Fig. 1). To better understand the evolutionary fixation dynamics on temporal networks, we introduce the concept of fixation time on each subnetwork, with the subnetworks occurring sequentially in time order and comprising the entire temporal network. We find that both synthetic and empirical heterogeneous temporal networks can favour the fixation of cooperative behaviour more than the homogeneous counterparts, which provides a unifying understanding of the evolution of cooperation across different theoretical frameworks. We further uncover the underlying mechanism of this advantage: heterogeneous temporal networks cause rapid strategy assortment over finite evolutionary timescales on subnetworks before structural switching, which in turn leads to the final evolutionary success.

## Results

We consider the evolutionary game dynamics on temporal networks composed of a sequence of subnetworks, where nodes indicate players and edges encode who interacts with whom (Fig. 1). Individuals choose either cooperation or defection. A cooperator pays a cost *c* to provide a benefit *b* to all of its opponents, and defectors provide no benefit and thus pay no cost. In each round, every player *i* interacts with all its neighbours separately and accumulates the obtained payoffs. At the end of each round, an individual is randomly chosen for updating its strategy^12,17^, where the individual either imitates the strategy of its neighbour *j* with the probability proportional to the fitness of *j*, denoted by *F*_*j*_, or retains its strategy with the probability proportional to its own fitness. Here, the fitness of *j* is *F*_*j*_ = 1 + *δf*_*j*_, where *f*_*j*_ is the payoff of player *j* in the current round, and 0 < *δ* ≪ 1 specifies the weak intensity of selection from game payoff to the reproductive fitness of each strategy^21^.

Starting with a single cooperator randomly placed in a population with *N* − 1 defectors, the evolutionary process involves *g* rounds of games and strategy updates on each subnetwork. The process ends when all players become either cooperator or defector (Supplementary Fig. 1)^15^. The fixation probability of cooperation (*ρ*_*C*_) on networks is defined by the probability that a single cooperator takes over the entire population^12,20,21^. Note that 1/*N* is the fixation probability in the case of a neutral drift, i.e., when the invader has the same fitness as the resident, on both static and temporal networks (Supplementary Fig. 2). Therefore, cooperation is favoured to invade and replace the population of full defectors by natural selection when the fixation probability of cooperation exceeds that of the neutral case. In other words, the network structure is said to favour the evolutionary success of cooperation if *ρ*_*C*_ > 1/*N*.

### Heterogeneity in temporal networks promotes cooperation

**Fig. 2:**
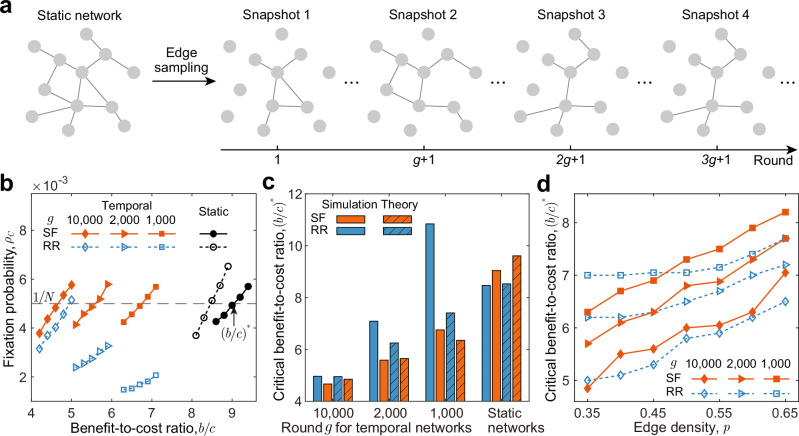
Comparison of the critical benefit-to-cost ratio for the fixation of cooperation between temporal scale-free (SF) networks and random regular (RR) networks. **a** We construct each snapshot by retaining the full set of nodes while randomly choosing part of the edges from an underlying static network. **b** For static networks, the critical benefit-to-cost ratio, (*b*/*c*)^*^, above which the fixation of cooperation is favoured, is higher for SF networks (indicated by the arrow pointing to the intersection of the black curve and the horizontal line) than the RR networks. For temporal SF networks (red markers), we find that (*b*/*c*)^*^ is lower than the temporal RR networks (blue markers) over different values of the round of interactions *g* on each snapshot. **c** Comparison between the numerical and theoretical results. **d** We further theoretically show that, when the edge density *p* is large, (*b*/*c*)^*^ is larger for temporal SF than that for RR networks. We generate synthetic temporal networks with 200 nodes and 100 subnetworks, and the static counterparts have an average degree of 6. We numerically calculate the fixation probability as the fraction of runs in which cooperators take over the whole population out of 4 × 10^5^ runs with the intensity of selection *δ* = 0.01.

Here we consider temporal networks as sequences of subnetworks sampled from an underlying static network. At every *g* generations of evolutionary dynamics, a subnetwork is generated by randomly selecting a fraction of edges from the underlying network, while the set of nodes remains unchanged (Fig. 2a). This random resampling framework allows us to model fluctuating interactions without altering the overall population structure. Importantly, we also consider alternative generative models, including node-rewiring dynamics and activity-driven networks, to ensure that our findings are robust across different construction methods (Supplementary Figs. 3 and 4). By comparing the fixation probabilities of cooperation under these different settings, we have confirmed that the main conclusions are not specific to any single modelling choice.

We start with the fixation dynamics of cooperation on static networks, including the scale-free network—a representative heterogeneous network with power-law degree distribution, and the random regular network with the same average degree as its counterparts. In line with previous results, Fig. 2b shows that the static scale-free networks present a higher critical benefit-to-cost ratio (*b*/*c*)^*^ than the static random regular networks (see filled and empty circles), where (*b*/*c*)^*^ is the threshold above which cooperation is favoured^12,21^, namely, *ρ*_*C*_ > 1/*N*. This provides the basis for the known conclusion that network heterogeneity hinders the evolution of cooperation^21,27^ (Supplementary Notes 1 and 2).

We next study the fixation of cooperation on temporal networks, wherein each subnetwork is generated by randomly activating a fraction of edges in the underlying static network, correspondingly (Fig. 2a). In this way, the temporal scale-free network not only shows a power-law distribution on the aggregated static network, but also exhibits strong degree heterogeneity in each of its subnetworks compared to temporal random regular networks. Figure 2b shows the fixation probability through the benefit-to-cost ratio on temporal scale-free and random regular networks. Surprisingly, we find that over different evolutionary timescales *g*, the temporal scale-free networks all have the significant advantage of facilitating the emergence of cooperation with a higher fixation probability than the temporal random regular networks. Note that this result is in sharp contrast with the previous conclusion drawn from comparing static scale-free and random regular networks^12,21,27^.

To theoretically understand the fixation of cooperation on temporal networks, we develop an analytical framework. We denote the probability of having *n* cooperators at the beginning of the *m*th subnetwork by *p*_*m*_(*n*), and describe the state of the system by a probability vector ${{{{\boldsymbol{p}}}}}_{m}={({p}_{m}(0),{p}_{m}(1),\ldots,{p}_{m}(N))}^{\top }$ with ${\sum }_{n=0}^{N}{p}_{m}(n)=1$, where ^⊤^ represents the transposition. Since each snapshot is dominated by a largest connected component (Supplementary Fig. 5), we consider the evolutionary dynamics on the largest connected component with *N*_*m*_ players of the *m*th snapshot. We denote by ${{{{\mathcal{T}}}}}_{m}^{g}(l,h)$ the transition probability starting from *l* cooperators in the largest connected component and ending with *h* cooperators after *g* rounds of evolution in the *m*th snapshot (see Methods). The state of the system between two successive subnetworks *m* and *m* + 1 obeys the master equation

1 $${p}_{m+1}(s)={\sum }_{n-l+h=s}{p}_{m}(n){q}_{m}(n,l){{{{\mathcal{T}}}}}_{m}^{g}(l,h),$$

where *q*_*m*_(*n*, *l*) captures the probability that there are *l* cooperators in the largest connected component of *N*_*m*_ players with *n* cooperators initially in the *m*th snapshot. And the fixation probability of cooperation on temporal networks is thus ${\rho }_{C}={lim}_{m\to \infty }{p}_{m}(N)$ starting from a single cooperator initially, and the critical threshold (*b*/*c*)^*^ is obtained as the value of (*b*/*c*), at which *ρ*_*C*_ is equal to 1/*N*. By theoretically calculating (*b*/*c*)^*^ on temporal networks with different evolutionary timescale *g* accordingly, we further confirm the tendency of a lower critical threshold for promoting cooperation on temporal scale-free networks with our theoretical framework (Fig. 2c).

Intuitively, when constructing a temporal network by activating a fraction *p* of the edges in each subnetwork, a higher *p* results in subnetworks that increasingly resemble a static network over time. For the sparse temporal networks, namely edges are activated with a relatively low fraction *p* of activation (e.g., *p* = 0.3), the difference in value of fixation probability between temporal scale-free and random regular networks is shrinking as the evolutionary timescale *g* increases (Fig. 2b). Interestingly, we demonstrate that, as the subnetworks become denser by increasing *p*, a turning point p^*^ of the advantage emerges, above which temporal random regular networks show lower threshold for promoting cooperation, aligning with previous results obtained for static networks (Fig. 2d, Supplementary Figs. 6 and 7). We find that the longer the evolutionary timescale is, the lower the value of the turning point p^*^ is, and temporal random regular networks can outperform temporal scale-free networks when *p* > p^*^ (Fig. 2d). For example, at *p* = 0.7, similar to the static scenario, the temporal random regular network performs better than the temporal scale-free network at fostering cooperation both theoretically and numerically (Supplementary Fig. 8).

To assess the generality of our results for a broader class of dynamic network models, we implemented a rewiring model in which, after every *g* evolutionary steps, a fraction *p*_*N*_ of nodes is selected, and each of their links is rewired with probability *p*_*E*_ to another randomly chosen node. We calculated the fixation probability of cooperation across a range of parameter settings for this rewiring model starting from a scale-free network, and compared the results with their homogeneous counterparts (Supplementary Fig. 3). We confirm our main conclusion—heterogeneous temporal networks can achieve an advantage in promoting cooperation, but this advantage gradually diminishes as the timescale *g* increases.

In addition, we considered an activity-driven model in which nodes are assigned activity potentials drawn from a power-law distribution^37^, and at each time step, active nodes form temporary connections with randomly chosen peers. Simulations on this model show results consistent with those obtained from both our proposed random edge sampling model and the rewiring model (Supplementary Fig. 4). In particular, for sparse networks, the advantage of heterogeneous temporal networks in facilitating cooperation diminishes as *g* increases. Yet for dense networks, the homogeneous networks outperform the heterogeneous temporal networks in promoting the fixation of cooperation across all values of *g* examined. These results further support the robustness and generality of our findings across different temporal network modelling approaches.

Moreover, we show that this general conclusion can be applied to different game types. We calculate the fixation probability on synthetic temporal scale-free networks compared to their homogeneous counterpart under snowdrift games (*T* = *β*, *R* = *β* − 1/2, *S* = *β* − 1, *P* = 0), and find that the advantage of heterogeneous networks shrinks when the networks become dense (Supplementary Fig. 9). This result further supports the robustness of our findings across different game types.

### Evolutionary dynamics on empirical temporal networks

**Fig. 3:**
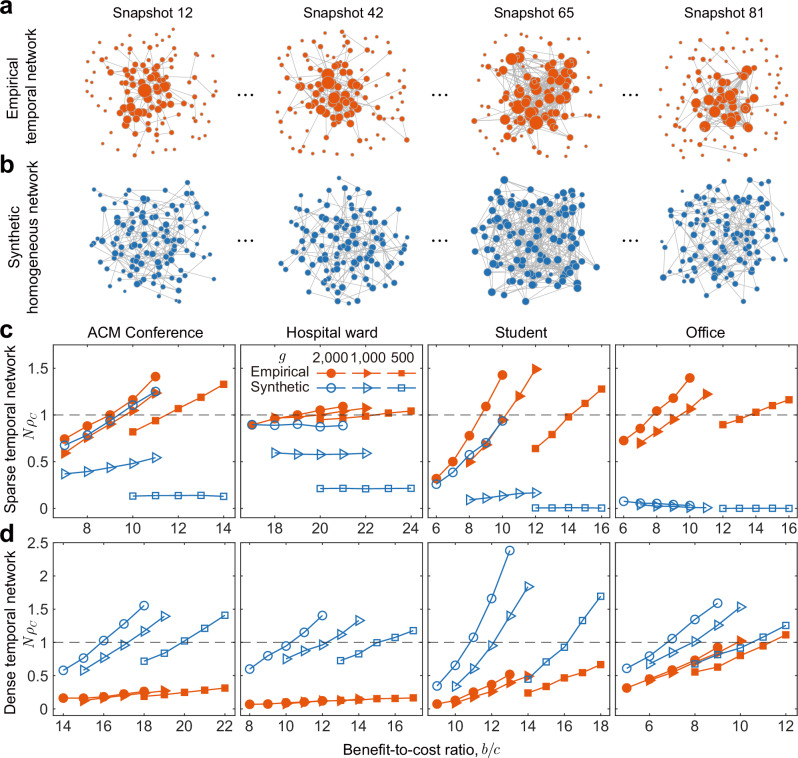
Fixation probability of cooperation on empirical temporal networks and the corresponding synthetic homogeneous networks. **a** Construction of empirical temporal networks based on the dataset capturing face-to-face proximity of 113 conference attendees over about 2.5 days in the ACM conference^38^. We obtain the sequence of subnetworks by aggregating interactions during the time interval *Δt* = 30 min. **b** Subnetworks in synthetic homogeneous temporal networks are generated with the same number of edges as that of the corresponding snapshot in the empirical temporal networks. **c** For four empirical datasets, i.e., ACM conference^38^, Hospital ward^39^, Student^40^ and Office^41^, we numerically show that sparse empirical temporal networks (*Δt* = 30 min, 30 min, 2 h, 2 h) yield a higher fixation probability (red markers) than the corresponding homogeneous temporal networks (blue markers), while the opposite holds true for dense empirical temporal networks (*Δt* = 6 h, 6 h, 12 h, 12 h) as shown in **d**. The details of the empirical dataset are presented in Supplementary Table 1 and Figs. 10-12.

Based on our findings on the evolution of cooperation on synthetic temporal networks, here we further extend our conclusions to a wider range of empirical networks. We construct temporal networks with empirical data collected from contacts in conference^38^, hospital^39^, high school^40^ and office workplace^41^, respectively, where each subnetwork is constructed from aggregating interactions in a fixed width of time window *Δt* (Supplementary Note 3, Supplementary Figs. 10 and 11). The larger width of the time window *Δt* is, the more interactions are involved in the subnetwork and the higher average degree of the network is. We show that both the subnetworks of empirical temporal networks and the aggregated static networks have relatively high degree heterogeneity (Fig. 3a, Supplementary Fig. 12).

To show whether empirical temporal networks can promote the emergence of cooperation compared to the homogeneous counterparts, we construct the corresponding homogeneous temporal network as a null model (Supplementary Fig. 13). Here each subnetwork in the homogeneous temporal networks has exactly the same number of edges as the original empirical subnetwork, while keeping each node with approximately the same number of neighbours (Fig. 3b). Specifically, we find that, for different values of *g*, cooperation fixates with a higher probability for empirical temporal networks than the homogeneous counterparts when the subnetworks are sparse for all empirical dataset (Fig. 3c). Similar to the results we find in the synthetic temporal scale-free networks, the advantage of the empirical heterogeneous temporal networks shrinks as the evolutionary timescale *g* increases (Fig. 3c). When the time window *Δt* becomes longer, we obtain the empirical networks with a higher average degree on each subnetwork, where the homogeneous counterparts present a higher fixation probability, therefore outperform the heterogeneous temporal networks in promoting cooperation (Fig. 3d). All these results are consistent with the numerical and theoretical findings on synthetic temporal networks shown in Fig. 2.

### Trade-off between fixation probability and fixation time

To intuitively understand the transition point in terms of the edge density we observed in both synthetic and empirical temporal networks, we now consider the case in which each subnetwork is connected so that *N*_*m*_ = *N* on each snapshot, and analyse the fixation probability of cooperation on temporal networks at *g* = 10^4^. The probability of reaching full cooperators (${{{{\mathcal{T}}}}}_{m}^{g}(l,N)$) or defectors (${{{{\mathcal{T}}}}}_{m}^{g}(l,0)$) starting from *l* cooperators is then fully determined by the fixation probability *ρ*^Sub^ and the expected strategy dispersal time *τ*^Sub^ on each subnetwork (see Methods). To understand *τ*^Sub^ in evolutionary dynamics, we calculate the average time 〈*t*^Sub^〉 that a single cooperator requires to take over the entire population on each subnetwork, namely the fixation time. Based on Eq. (11), for large networks, we obtain

2 $$\langle {t}^{{{{\rm{Sub}}}}}\rangle=\frac{1}{{\rho }^{{{{\rm{Sub}}}}}}{\sum }_{g=1}^{\infty }g\left({{{{\mathcal{T}}}}}_{m}^{g}(1,N)-{{{{\mathcal{T}}}}}_{m}^{g-1}(1,N)\right) \sim \frac{N{\tau }^{{{{\rm{Sub}}}}}}{2}.$$

In other words, the expected strategy dispersal time *τ*^Sub^ is approximately proportional to the fixation time of cooperation on each snapshot 〈*t*^Sub^〉. For ease of understanding, we now consider that each subnetwork generally presents the same fixation probability *ρ*^Sub^ and the expected strategy dispersal time *τ*^Sub^, and the probability distribution of the number of cooperators on temporal networks as *m* tends to infinity is

3 $${lim}_{m\to \infty }{{{{\boldsymbol{p}}}}}_{m}^{\top }={{{{\boldsymbol{p}}}}}_{0}^{\top }{\prod }_{m=0}^{\infty }{{{{\mathcal{T}}}}}_{m}^{g}.$$

**Fig. 4:**
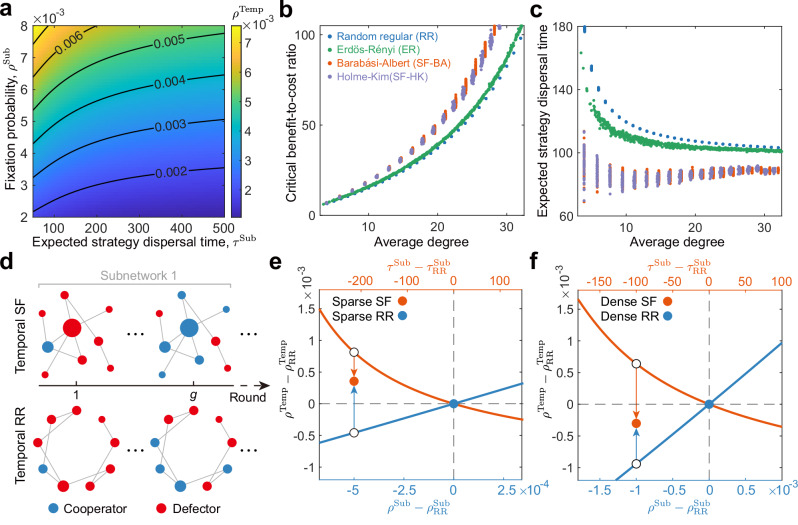
The fixation probability and fixation time on each subnetwork combine to determine the fixation dynamics on temporal networks. **a** We theoretically show the contour of the fixation probability of cooperation on temporal networks (*ρ*^Temp^) as a function of the fixation probability (*ρ*^Sub^) and strategy dispersal time (*τ*^Sub^) on each subnetwork under evolutionary timescale *g* = 10^4^. We present the critical threshold (*b*/*c*)^*^ in **b** and expected strategy dispersal time in **c** on four typical network structures: random regular (RR), Erdös-Rényi (ER), Barabasi-Albert (SF-BA) and Holme-Kim (SF-HK) scale free over different average degrees. **d** We illustrate the fundamental advantage of temporal scale-free (SF) networks during the course of evolution: highly heterogeneous subnetworks allow the fast formation of cooperative clusters in finite rounds *g*, which facilitates the further dispersal of cooperation in the subsequent subnetworks. In contrast, homogeneous subnetworks require a longer time for strategy dispersal, thus leading to less progress in strategy dispersal before structural changes. **e** By setting the fixation probability (${\rho }_{\,{{{\rm{RR}}}}}^{{{{\rm{Sub}}}}}$) and dispersal time (${\tau }_{\,{{{\rm{RR}}}}}^{{{{\rm{Sub}}}}}$) on the subnetwork of temporal RR networks as the baseline, we present how the alteration of *ρ*^Sub^ and *τ*^Sub^ changes the fixation probability *ρ*^Temp^ on a temporal network. Specifically, relative to the case for a sparse temporal RR network (*p* = 0.3, blue solid dot, ${\rho }^{{{{\rm{Temp}}}}}={\rho }_{{{{\rm{RR}}}}}^{{{{\rm{Temp}}}}}$), a sparse temporal SF network (red solid dot, which is obtained by the summation of two white solid dots) yields the change in *ρ*^Temp^ caused by ${\tau }^{{{{\rm{Sub}}}}}-{\tau }_{{{{\rm{RR}}}}}^{{{{\rm{Sub}}}}}$ (red line) being larger than the change caused by ${\rho }^{{{{\rm{Sub}}}}}-{\rho }_{{{{\rm{RR}}}}}^{{{{\rm{Sub}}}}}$ (blue line). This combined effect increases the corresponding fixation probability on sparse temporal SF networks. **f** In dense networks (*p* = 0.7), the increase in *ρ*^Temp^ caused by ${\tau }^{{{{\rm{Sub}}}}}-{\tau }_{{{{\rm{RR}}}}}^{{{{\rm{Sub}}}}}$ (red line) does not compensate the decrease in *ρ*^Temp^ caused by ${\rho }^{{{{\rm{Sub}}}}}-{\rho }_{{{{\rm{RR}}}}}^{{{{\rm{Sub}}}}}$ (blue line), leading to the smaller fixation probability for temporal SF than RR networks (red solid dot). Other parameters are the same as those in Fig 2.

We now uncover the trade-off between fixation probability and fixation time on subnetworks during the course of the evolution on temporal networks. Figure 4a shows that a shorter strategy dispersal time (*τ*^Sub^) may sustain the same fixation probability of cooperation on temporal networks (*ρ*^Temp^) at a lower fixation probability on each subnetwork (*ρ*^Sub^). In other words, the disadvantage of fixation probability for subnetworks can actually be compensated by the fast strategy dispersal within the finite evolutionary timescale *g*. With this in mind, we present the critical threshold which captures the ability of fixation (Fig. 4b), and also the strategy dispersal time representing the speed of fixation (Fig. 4c), for typical network structures (e.g. scale-free, Erdös-Rényi, random regular) over different average degrees. We show that scale-free networks always require a higher critical threshold to promote cooperation, which indicates a lower fixation probability at the same *b*/*c* compared to homogeneous networks (Fig. 4b). However, networks with high degree heterogeneity spread the strategy fast due to the existence of hub nodes, which results in a shorter fixation time (Fig. 4c).

We now uncover the underlying mechanism of why temporal scale-free networks favour the fixation of cooperation more than the temporal random regular networks in the case of sparse subnetworks. Specifically, despite that the fixation probability for static scale-free networks with low average degree is slightly lower than that for static random regular networks (Fig. 4b), the former has a significantly shorter strategy dispersal time (Fig. 4c), which facilitates the emergence of cooperative clusters in each snapshot at finite evolutionary timescale, *g* (Fig. 4d). In contrast, the static random regular networks require a much longer time for fixation, making it difficult for cooperators to diffuse and cluster at finite *g* (Fig. 4d). Therefore, sparse temporal scale-free networks can achieve a larger fixation probability than sparse temporal random regular networks by taking advantage of shorter strategy dispersal time on each snapshot (red dot in Fig. 4e). As subnetworks become denser, the difference in the fixation probability between random regular and scale-free subnetworks increases drastically yet the difference between fixation time further shrinks (Fig. 4b, c). Then, the disadvantage from the fixation probability for the static scale-free networks can not be compensated with the shorter fixation time (red dot in Fig. 4f). This trade-off effect between the fixation probability and fixation time is also confirmed in empirical temporal networks (Fig. 3c, d).

### Effect of temporal evolutionary timescales

Finally, we explore effects of the evolutionary timescale *g* on each subnetwork. In general, a larger *g* allows the emergence and preservation of clusters of cooperators before the switching of subnetworks, resulting in a higher fixation probability for cooperation, as shown in Figs. 2 and 3. The fixation probability on temporal networks in the limit *g* → *∞* is determined solely by the fixation probability on the first subnetwork, namely a single static network. Moreover, the ratio between the evolutionary timescale *g* and the strategy dispersal time *τ*^Sub^ jointly determines the evolutionary progression on subnetworks. In this way, for the same evolutionary timescale *g*, networks with shorter strategy dispersal time lead to a higher completion of strategy assortment and fixation, which provides the basis for the advantage of temporal scale-free networks. On the other hand, as *g* increases, the advantage on favouring cooperation brought by the shorter fixation time is discounted since different network structures all tend to complete the evolutionary process, so that the critical benefit-to-cost ratio decreases monotonically with the evolutionary timescale *g*. This discounted effect for promoting cooperation on heterogeneous temporal networks from increasing evolutionary timescale *g* is confirmed in both synthetic and empirical temporal networks (Figs. 2 and 3). While our simulations suggest a generally monotonic effect of *g* in snapshot-based temporal network models, whether such monotonicity holds on arbitrary temporal networks remains an open question.

Although so far we have mainly concerned with situations where evolution is much faster than changes in network structure, namely large evolutionary timescales on each subnetwork, there are also scenarios with equivalent or faster structural changes relative to the evolutionary dynamics. And instances may include electronic communication with very short time resolution between intelligent agents. Here we capture these cases with *g* = 1 or even *g* < 1, and we confirm that our main result still holds qualitatively (Supplementary Fig. 14). Specifically, when the networks are sparse, temporal scale-free networks outperform temporal random regular networks for promoting the fixation of cooperation (Supplementary Fig. 14a), yet the trend reverses when networks become denser (Supplementary Fig. 14b). This phenomenon is exactly the same as that with large evolutionary timescales.

Moreover, we further consider a mixed temporal network model, where a fraction *p*_*s*_ of each evolutionary period is constructed from scale-free networks and the remaining fraction 1 − *p*_*s*_ from random regular networks, with the two types alternating in successive periods (Supplementary Fig. 15). Compared to the case in which the subnetworks are drawn from a single static substrate network, these mixed temporal networks consistently yield the lowest fixation probability of cooperation across different evolutionary timescales. Mechanistically, this suppression effect can be attributed to the disruption of structural similarity. While subnetworks sampled from the same underlying network preserve certain topological correlations that support the sustaining of cooperative clusters, alternating between fundamentally different network types undermines such correlations, making the emergence of cooperation more difficult.

## Discussion

With the rise of network science, researchers have increasingly focused on the impact of dynamic networks on the evolution of cooperation^15,42–44^. While previous study has modelled dynamic systems using stochastic networks with predefined transition probabilities^42^, we take an alternative approach by directly generating temporal networks from empirical datasets, based on sequences of interactions. To capture the interplay between the speed of strategy evolution and structural changes, we introduce the finite evolutionary timescale on each snapshot. Our analyses reveal that heterogeneous temporal networks can foster cooperation more effectively than homogeneous temporal networks across various modelling approaches. Provided the fact that static heterogeneous networks inhibit the emergence of cooperation compared to their homogeneous counterparts in fixation dynamics when imitation updates are adopted^10,12,45^, it is striking that heterogeneous temporal networks can offset this disadvantage through shorter fixation time across subnetworks.

Adopting the perspective of fixation probability in temporal networks, we derive conclusions that align with those obtained in the framework of the equilibrium frequency of cooperators on static networks. It is worth noting that our temporal network framework is different from another important class of dynamic networks in previous studies—the coevolutionary dynamics where the network structure and individual strategies evolve simultaneously^16,18,46^. In our study, whether considering real-world or artificially generated temporal networks, the network structure evolves independently of the game dynamics—our focus is on how the temporal evolution of the network affects strategy dynamics. This exogenous nature of network evolution, where strategy updates do not affect the network structure, allows for a more specific investigation of how network dynamics at different timescales affect the emergence of collective behaviour. Similar approaches could be extended to explore other dynamical processes, such as epidemic spreading^32^ and opinion dynamics^47^.

Different network models capture different aspects of reality. The random resampling models can describe situations where interactions are randomly drawn in each period. The activity-driven model also draws interactions randomly on top of an effective substrate network but emphasises random node activations. Rewiring models are more appropriate for systems in which most interactions persist but occasionally change. While these models highlight different empirical scenarios, our main conclusions remain valid across synthetic models and empirical datasets (Figs. 2 and 3, Supplementary Figs. 3 and 4). Future work may further integrate synthetic frameworks and empirical systems through hybrid or data-driven temporal models.

In traditional studies of switching systems, average systems are often used for deriving stability or instability criteria^36,48^, and a similar question arises in our context of cooperation on temporal networks. Previous work has shown that when cooperators randomly select a recipient of a donation at each time step, the final evolutionary outcome is equivalent to a static network where contributions are evenly distributed among all possible recipients under weak selection^49^. However, previous studies have shown that when the evolution of network structures is involved, the collective dynamics cannot be effectively approximated by averaging methods^50,51^. For temporal networks—where structural changes are exogenous and often exhibit bursty rhythms—it remains an open challenge to identify effective averaging approaches. We believe that our conclusions may provide a useful foundation for developing such methods in the future.

A natural implication is how fixation time plays a role in the evolutionary dynamics on temporal networks. There is a large body of research analysing the fixation probability on static network structures^10,12,21,45^, yet relatively few studies have focused on the fixation time^52,53^. Here we uncover that the advantage of temporal scale-free networks actually roots in the short fixation time on each subnetwork, which allows the fast formation of cooperative clusters before structural switching. This indicates that the fixation time of the structure, together with the fixation probability, jointly determine the temporal evolutionary dynamics with finite evolutionary timescale. Our findings open the door to exploring evolutionary dynamics with a broader perspective, and pave the way for future investigations on temporal networks underlying realistic complex systems.

## Methods

### Cooperative dynamics on subnetworks with finite timescale

We first consider the cooperative dynamics with finite rounds of evolution on subnetworks. In each round of games, individuals play the donation games with their immediate neighbours and accumulate the payoffs accordingly on the connected component of subnetworks, where *w*_*ij*_ = *w*_*ji*_ captures the edge weights on the largest component. The payoff matrix of the game is given by

4 $$\begin{array}{l}\,\,\,\,\,\,\,\,\,{{{\rm{C}}}}\,\,\,\,\,\,\,\,\,\,\,\,\,{{{\rm{D}}}}\\ \begin{array}{l}\,{{{\rm{C}}}}\\ \,{{{\rm{D}}}}\end{array}\left(\begin{array}{cc}b-c & -c\\ b & 0\end{array}\right).\end{array}$$

And the state of the evolutionary process on the connected component with *N*_*m*_ nodes is given by a binary vector ${{{\bf{x}}}}\in {\{0,1\}}^{{N}_{m}}$, where *x*_*i*_ = 1 denotes that the player *i* chooses strategy C, otherwise *x*_*i*_ = 0 indicates strategy D. Using the imitation dynamics where individuals imitate the strategy or keep its own strategy according to the corresponding fitness, we have the probability that *i* copies *j*’s strategy in state **x** is

5 $${e}_{ji}({{{\bf{x}}}})=\frac{1}{{N}_{m}}\frac{{\widetilde{w}}_{ij}{F}_{j}({{{\bf{x}}}})}{{\sum }_{l=1}^{{N}_{m}}{\widetilde{w}}_{il}{F}_{l}({{{\bf{x}}}})},$$

where ${\widetilde{w}}_{ij}$ defines the replacement networks under imitation dynamics capturing who imitates the strategy from whom, where ${\widetilde{w}}_{ii}=1$ and ${\widetilde{w}}_{ij}={w}_{ij}$ if *i* ≠ *j* (Supplementary Note 1). Following the evolution of games and updates over finite round *g*, the weighted average frequency of cooperators is given by

6 $${{\mathbb{E}}}_{{{{\bf{u}}}}}[\widehat{x}(g)]=\frac{l}{{N}_{m}}+\delta \mathop{\sum }_{t=0}^{g-1}{{\mathbb{E}}}_{{{{\bf{u}}}}}^{\circ }\left[{\left.\frac{{{{\rm{d}}}}D\left({{{\bf{X}}}}(t)\right)}{{{{\rm{d}}}}\delta }\right|}_{\delta=0}\right]+{{{\mathcal{O}}}}\left({\delta }^{2}\right),$$

where $\widehat{x}(g)={\sum }_{i}{\widetilde{\pi }}_{i}{x}_{i}(g)$, where ${\widetilde{\pi }}_{i}$ is the reproductive value^54,55^ uniquely solved by Supplementary equation (2) and the subscript **u** indicates the uniform initialisation of cooperators. Here $D({{{\bf{x}}}}):={\mathbb{E}}\left[\widehat{x}(t+1)-\widehat{x}(t)| {{{\bf{X}}}}(t)={{{\bf{x}}}}\right]$ defines the expected change of $\widehat{x}$ from state **x**, and we have

7 $${\left.\frac{{{{\rm{d}}}}D({{{\bf{x}}}})}{{{{\rm{d}}}}\delta }\right|}_{\delta=0}={\sum }_{i=1}^{N}{\widetilde{\pi }}_{i}{\sum }_{j=1}^{N}({x}_{j}-{x}_{i}){\left.\frac{{{{\rm{d}}}}{e}_{ji}({{{\bf{x}}}})}{{{{\rm{d}}}}\delta }\right|}_{\delta=0}.$$

Here ${\widetilde{\pi }}_{i}=({\sum }_{j}{w}_{ij}+1)/({\sum }_{i,j}{w}_{ij}+{N}_{m})$ indicates the reproductive rate^55^ of node *i*.

### Modelling the switching system

We develop an analytical framework to model the switching system by the transition probability ${{{{\mathcal{T}}}}}_{m}^{g}$, where ${{{{\mathcal{T}}}}}_{m}^{g}(l,h)$ captures the probability that the system starts from *l* cooperators in the largest connected component in the *m*th snapshot and reaches *h* cooperators after *g* rounds of evolution. To approximate ${{{{\mathcal{T}}}}}_{m}^{g}(l,h)$, we consider the weighted average frequency of cooperators at the *g*th round, ${{\mathbb{E}}}_{{{{\bf{u}}}}}\left[\widehat{x}(g)\right]$, on the largest component in the *m*th snapshot. Specifically, we have *w*_*i*_ = ∑_*j*_*w*_*ij*_ indicating the weighted degree for node *i*. Based on the cooperative dynamics with finite evolutionary timescales, we have

8 $${{\mathbb{E}}}_{{{{\bf{u}}}}}\left[\widehat{x}(g)\right]=\frac{l}{{N}_{m}}+\frac{l({N}_{m}-l)\delta }{2{N}_{m}({N}_{m}-1)}\left[-c{\sum }_{i,j}{\widetilde{\pi }}_{i}{\widetilde{p}}_{ij}^{(2)}{w}_{j}{\widetilde{\tau }}_{ij}\left(T\right)\right.\\+b\left.\left({\sum }_{i,j,k}{\widetilde{\pi }}_{i}{\widetilde{p}}_{ij}^{(2)}{w}_{j}{p}_{jk}{\widetilde{\tau }}_{ik}\left(T\right)-{\sum }_{i,j}{\widetilde{\pi }}_{i}{w}_{i}{p}_{ij}{\widetilde{\tau }}_{ij}\left(T\right)\right)\right],$$

where *p*_*ij*_ is the probability of a single step random walk from *i* to *j* on the connected component; ${\widetilde{p}}_{ij}^{(n)}$ is the probability of an *n*-step random walk on the corresponding replacement network; P_{*i*, *j*}_(*τ*) represents the probability that two random walkers (one starting at node *i* and the other at node *j*) coalesce at time *τ* (Supplementary Note 1); ${\widetilde{\tau }}_{ij}(T)={\sum }_{t=0}^{T}{\sum }_{\tau=t+1}^{\infty }{{{{\rm{P}}}}}_{\{i,j\}}(\tau )$ represents the accumulation of the probability that *i* and *j* have not coalesced until *t*. Here *T* ≡ ⌊2(*g* − 1)/*N*_*m*_⌋ represents the effective *g*, rescaled by the probability 2/*N*_*m*_ that the individual chosen for updating is one of the two random walkers (Supplementary Note 1). Specifically, P_{*i*, *j*}_(*τ*) *ijτ* follows the recurrence relations given by

9 $${{{{\rm{P}}}}}_{\{i,j\}}(\tau )=\left\{\begin{array}{ll}\frac{1}{2}{\sum }_{k=1}^{N}{\widetilde{p}}_{ik}{{{{\rm{P}}}}}_{\{k,j\}}(\tau -1)+\frac{1}{2}{\sum }_{k=1}^{N}{\widetilde{p}}_{jk}{{{{\rm{P}}}}}_{\{k,i\}}(\tau -1),& \!{{{\rm{if}}}}\,\,i\ne j\\ 0,\hfill& \,{{{\rm{if}}}}\,\,i=j\end{array}\right.$$

when *τ* > 0. When *τ*=0, we have

10 $${{{{\rm{P}}}}}_{\{i,j\}}(0)=\left\{\begin{array}{ll}0 & \!\!\!{{{\rm{if}}}}\,\,i\ne j\\ 1 & \,{{{\rm{if}}}}\,\,i=j.\end{array}\right.$$

### Evaluating the fixation probability on temporal networks

Based on the probability distribution of the number of cooperators starting from *l* cooperators after *g* rounds of evolution we observed numerically in Supplementary Fig. 16, we theoretically write the transition probability as

11 $$	{{{{\mathcal{T}}}}}_{m}^{g}(l,0) \approx \left(1-{{\mathrm{lim}}}_{g^{\prime} \to \infty }{{\mathbb{E}}}_{{{{\bf{u}}}}}\left[\widehat{x}(g^{\prime} )\right]\right)\left[1-\exp \left(\frac{2(1-{N}_{m})g}{l{N}_{m}{\tau }_{m}}\right)\right],\\ 	 {{{{\mathcal{T}}}}}_{m}^{g}(l,{N}_{m}) \approx {{\mathrm{lim}}}_{g^{\prime} \to \infty }{{\mathbb{E}}}_{{{{\bf{u}}}}}\left[\widehat{x}(g^{\prime} )\right]\left[1-\exp \left(\frac{2(1-{N}_{m})g}{({N}_{m}-l){N}_{m}{\tau }_{m}}\right)\right],\\ 	 {{{{\mathcal{T}}}}}_{m}^{g}(l,h) \approx \alpha {\beta }^{| h-l| }\,(0 < h < {N}_{m}),$$

where *τ*_*m*_ is the expectation of the coalescence time^56^ averaged over the component of *N*_*m*_ players in the snapshot *m*, which we note for the strategy dispersal time *τ*^Sub^ on subnetworks for generalizations. When *l* = 1, ${{\mathrm{lim}}}_{g^{\prime} \to \infty }{{\mathbb{E}}}_{{{{\bf{u}}}}}\left[\widehat{x}(g^{\prime} )\right]$ is equivalent to the fixation probability of cooperation on the component. Based on the definition of the transition probability, we obtain the above parameters *α* and *β* by imposing

12 $${\sum }_{h=0}^{{N}_{m}}{{{{\mathcal{T}}}}}_{m}^{g}(l,h)=1$$

and

13 $$\mathop{\sum }_{h=0}^{{N}_{m}}\frac{h}{{N}_{m}}{{{{\mathcal{T}}}}}_{m}^{g}(l,h)\approx {{\mathbb{E}}}_{{{{\bf{u}}}}}\left[\widehat{x}(g)\right].$$

Under the approximation that cooperators are uniformly distributed at the onset of each snapshot, the probability that there are *l* cooperators in the largest connected component of *N*_*m*_ players is $${q}_{m}(n,l)=\left(\begin{array}{l}n\\ l\end{array}\right)\left(\begin{array}{l}N-n\\ {N}_{m}-l\end{array}\right)/\left(\begin{array}{l}N\\ {N}_{m}\end{array}\right)$$. By applying numerical iterations based on the master equation given in Eq. (1), we obtain the fixation probability on temporal networks and the critical threshold (*b*/*c*)^*^ accordingly. We show that our theoretical results remain valid across a wide range of edge density *p*, even in the case that the subnetworks are relatively sparse (Supplementary Fig. 7).

### Reporting summary

Further information on research design is available in the Nature Portfolio Reporting Summary linked to this article.

## Supplementary information

Supplementary Information

Reporting Summary

Transparent Peer Review file

## Data Availability

All data generated in this study are obtained from numerical simulations and are included within the paper and its supplementary information files. The empirical network datasets used in Fig. 3 (ACM conference^38^, Hospital ward^39^, Student^40^, and Office^41^ contact) are publicly available through the SocioPatterns collaboration (http://www.sociopatterns.org), and can be accessed through the corresponding Refs. 38-41.

## Code availability

The codes are written using MathWorks MATLAB R2021a and Python 3.8.5. All source codes related to the work can be found at^57^https://github.com/yaomeng1/Temporality.

## References

[1] Hofbauer, J. & Sigmund, K.*Evolutionary Games and Population Dynamics* (Cambridge Univ. Press, 1998).

[2] Rapoport, A., Chammah, A. M. & Orwant, C. J.*Prisoner’s Dilemma: A Study in Conflict and Cooperation* (Michigan Univ. Press, 1965).

[3] Lieberman, E., Hauert, C. & Nowak, M. A. Evolutionary dynamics on graphs. *Nature* **433**, 312–316 (2005). 15662424 10.1038/nature03204

[4] Taylor, P. D., Day, T. & Wild, G. Evolution of cooperation in a finite homogeneous graph. *Nature* **447**, 469–472 (2007). 17522682 10.1038/nature05784

[5] Nowak, M. A. & May, R. M. Evolutionary games and spatial chaos. *Nature* **359**, 826–829 (1992).

[6] Hauert, C. & Doebeli, M. Spatial structure often inhibits the evolution of cooperation in the snowdrift game. *Nature* **428**, 643–646 (2004). 15074318 10.1038/nature02360

[7] Hilbe, C., Šimsa, Š, Chatterjee, K. & Nowak, M. A. Evolution of cooperation in stochastic games. *Nature* **559**, 246–249 (2018). 29973718 10.1038/s41586-018-0277-x

[8] Santos, F. P., Santos, F. C. & Pacheco, J. M. Social norm complexity and past reputations in the evolution of cooperation. *Nature* **555**, 242–245 (2018). 29516999 10.1038/nature25763

[9] Colnaghi, M., Santos, F. P., Van Lange, P. A. & Balliet, D. Adaptations to infer fitness interdependence promote the evolution of cooperation. *Proc. Natl. Acad. Sci. U.S.A.* **120**, e2312242120 (2023). 38055736 10.1073/pnas.2312242120 PMC10723045

[10] Meng, Y., Cornelius, S. P., Liu, Y.-Y. & Li, A. Dynamics of collective cooperation under personalised strategy updates. *Nat. Commun.* **15**, 3125 (2024). 38600076 10.1038/s41467-024-47380-8 PMC11006938

[11] Santos, F. C. & Pacheco, J. M. Scale-free networks provide a unifying framework for the emergence of cooperation. *Phys. Rev. Lett.* **95**, 098104 (2005). 16197256 10.1103/PhysRevLett.95.098104

[12] Ohtsuki, H., Hauert, C., Lieberman, E. & Nowak, M. A simple rule for the evolution of cooperation on graphs and social networks. *Nature* **441**, 502–505 (2006). 16724065 10.1038/nature04605 PMC2430087

[13] Bao, X., Hu, Q., Ji, P., Lin, W., Kurths, J. & Nagler, J. Impact of basic network motifs on the collective response to perturbations. *Nat. Commun.* **13**, 5301 (2022). 36075905 10.1038/s41467-022-32913-w PMC9458749

[14] Paré, P. E., Beck, C. L. & Başar, T. Modeling, estimation, and analysis of epidemics over networks: An overview. *Annu. Rev. Control* **50**, 345–360 (2020).

[15] Li, A., Zhou, L., Su, Q., Cornelius, S. P., Liu, Y.-Y., Wang, L. & Levin, S. A. Evolution of cooperation on temporal networks. *Nat. Commun.* **11**, 2259 (2020). 32385279 10.1038/s41467-020-16088-w PMC7210286

[16] Perc, M. & Szolnoki, A. Coevolutionary games—a mini review. *BioSystems* **99**, 109–125 (2010). 19837129 10.1016/j.biosystems.2009.10.003

[17] Perc, M., Gómez-Gardenes, J., Szolnoki, A., Floría, L. M. & Moreno, Y. Evolutionary dynamics of group interactions on structured populations: a review. *J. R. Soc. Interface* **10**, 20120997 (2013). 23303223 10.1098/rsif.2012.0997 PMC3565747

[18] Szolnoki, A., Perc, M. & Szabó, G. Topology-independent impact of noise on cooperation in spatial public goods games. *Phys. Rev. E* **80**, 056109 (2009). 10.1103/PhysRevE.80.056109 20365045

[19] Szolnoki, A., Perc, M. & Szabó, G. Defense mechanisms of empathetic players in the spatial ultimatum game. *Phys. Rev. Lett.* **109**, 078701 (2012). 23006406 10.1103/PhysRevLett.109.078701

[20] Nowak, M. A., Sasaki, A., Taylor, C. & Fudenberg, D. Emergence of cooperation and evolutionary stability in finite populations. *Nature* **428**, 646–650 (2004). 15071593 10.1038/nature02414

[21] Allen, B., Lippner, G., Chen, Y.-T., Fotouhi, B., Momeni, N., Yau, S.-T. & Nowak, M. A. Evolutionary dynamics on any population structure. *Nature* **544**, 227–230 (2017). 28355181 10.1038/nature21723

[22] Meng, Y., McAvoy, A. & Li, A. Promoting collective cooperation through temporal interactions. *Proc. Natl. Acad. Sci. U.S.A.* **122**, e2509575122 (2025). 40577118 10.1073/pnas.2509575122 PMC12232700

[23] Barabási, A.-L. & Albert, R. Emergence of scaling in random networks. *Science* **286**, 509–512 (1999). 10521342 10.1126/science.286.5439.509

[24] Newman, M. *Networks* (Oxford Univ. Press, 2018).

[25] Santos, F. C., Santos, M. D. & Pacheco, J. M. Social diversity promotes the emergence of cooperation in public goods games. *Nature* **454**, 213–216 (2008). 18615084 10.1038/nature06940

[26] Santos, F. C., Pacheco, J. M. & Lenaerts, T. Evolutionary dynamics of social dilemmas in structured heterogeneous populations. *Proc. Natl. Acad. Sci. U.S.A.* **103**, 3490–3494 (2006). 16484371 10.1073/pnas.0508201103 PMC1413882

[27] Fotouhi, B., Momeni, N., Allen, B. & Nowak, M. A. Evolution of cooperation on large networks with community structure. *J. R. Soc. Interface* **16**, 20180677 (2019). 30862280 10.1098/rsif.2018.0677 PMC6451403

[28] Gracia-Lázaro, C., Ferrer, A., Ruiz, G., Tarancón, A., Cuesta, J. A., Sánchez, A. & Moreno, Y. Heterogeneous networks do not promote cooperation when humans play a prisoner’s dilemma. *Proc. Natl. Acad. Sci. U.S.A.* **109**, 12922–12926 (2012). 22773811 10.1073/pnas.1206681109 PMC3420198

[29] Masuda, N. & Lambiotte, R. *A Guide to Temporal Networks* (World Scientific, 2020).

[30] Li, A., Cornelius, S. P., Liu, Y.-Y., Wang, L. & Barabási, A.-L. The fundamental advantages of temporal networks. *Science* **358**, 1042–1046 (2017). 29170233 10.1126/science.aai7488

[31] Holme, P. & Saramäki, J. Temporal networks. *Phys. Rep.* **519**, 97–125 (2012).

[32] Masuda, N., Klemm, K. & Eguíluz, V. M. Temporal networks: slowing down diffusion by long lasting interactions. *Phys. Rev. Lett.* **111**, 188701 (2013). 24237569 10.1103/PhysRevLett.111.188701

[33] Scholtes, I., Wider, N., Pfitzner, R., Garas, A., Tessone, C. J. & Schweitzer, F. Causality-driven slow-down and speed-up of diffusion in non-markovian temporal networks. *Nat. Commun.* **5**, 5024 (2014). 25248462 10.1038/ncomms6024

[34] Saavedra, S., Rohr, R. P., Fortuna, M. A., Selva, N. & Bascompte, J. Seasonal species interactions minimize the impact of species turnover on the likelihood of community persistence. *Ecology* **97**, 865–873 (2016). 27220203 10.1890/15-1013.1

[35] Kossinets, G. & Watts, D. J. Empirical analysis of an evolving social network. *Science* **311**, 88–90 (2006). 16400149 10.1126/science.1116869

[36] Allesina, S. & Tang, S. Stability criteria for complex ecosystems. *Nature* **483**, 205–208 (2012). 22343894 10.1038/nature10832

[37] Perra, N., Gonçalves, B., Pastor-Satorras, R. & Vespignani, A. Activity driven modeling of time varying networks. *Sci. Rep.* **2**, 469 (2012). 22741058 10.1038/srep00469 PMC3384079

[38] Isella, L., Stehlé, J., Barrat, A., Cattuto, C., Pinton, J.-F. & Van den Broeck, W. What’s in a crowd? analysis of face-to-face behavioral networks. *J. Theor. Biol.* **271**, 166–180 (2011). 21130777 10.1016/j.jtbi.2010.11.033

[39] Vanhems, P., Barrat, A., Cattuto, C., Pinton, J.-F., Khanafer, N., Régis, C., Kim, B. -a, Comte, B. & Voirin, N. Estimating potential infection transmission routes in hospital wards using wearable proximity sensors. *PLOS ONE* **8**, e73970 (2013). 24040129 10.1371/journal.pone.0073970 PMC3770639

[40] Fournet, J. & Barrat, A. Contact patterns among high school students. *PLOS ONE* **9**, e107878 (2014). 25226026 10.1371/journal.pone.0107878 PMC4167238

[41] Génois, M., Vestergaard, C. L., Fournet, J., Panisson, A., Bonmarin, I. & Barrat, A. Data on face-to-face contacts in an office building suggest a low-cost vaccination strategy based on community linkers. *Netw. Sci.* **3**, 326–347 (2015).

[42] Su, Q., McAvoy, A. & Plotkin, J. B. Strategy evolution on dynamic networks. *Nat. Comput. Sci.* **3**, 763–776 (2023). 38177777 10.1038/s43588-023-00509-z

[43] Johnson, T. & Smirnov, O. Temporal assortment of cooperators in the spatial prisoner’s dilemma. *Commun. Biol.* **4**, 1283 (2021). 34773077 10.1038/s42003-021-02804-9 PMC8589994

[44] Sheng, A., Li, A. & Wang, L. Evolutionary dynamics on sequential temporal networks. *PLoS Comput. Biol.* **19**, e1011333 (2023). 37549167 10.1371/journal.pcbi.1011333 PMC10434888

[45] Wang, X., Zhou, L., McAvoy, A. & Li, A. Imitation dynamics on networks with incomplete information. *Nat. Commun.* **14**, 7453 (2023). 37978181 10.1038/s41467-023-43048-x PMC10656501

[46] Pacheco, J. M., Traulsen, A. & Nowak, M. A. Coevolution of strategy and structure in complex networks with dynamical linking. *Phys. Rev. Lett.* **97**, 258103 (2006). 17280398 10.1103/PhysRevLett.97.258103 PMC2430061

[47] Ghaderi, J. & Srikant, R. Opinion dynamics in social networks with stubborn agents: Equilibrium and convergence rate. *Automatica* **50**, 3209–3215 (2014).

[48] Allesina, S., Grilli, J., Barabás, G., Tang, S., Aljadeff, J. & Maritan, A. Predicting the stability of large structured food webs. *Nat. Commun.* **6**, 7842 (2015). 26198207 10.1038/ncomms8842 PMC4525179

[49] McAvoy, A., Allen, B. & Nowak, M. A. Social goods dilemmas in heterogeneous societies. *Nat. Hum. Behav.* **4**, 819–831 (2020). 32451481 10.1038/s41562-020-0881-2

[50] Wu, B., Zhou, D., Fu, F., Luo, Q., Wang, L. & Traulsen, A. Evolution of cooperation on stochastic dynamical networks. *PLOS ONE* **5**, e11187 (2010). 20614025 10.1371/journal.pone.0011187 PMC2894855

[51] Traulsen, A., Claussen, J. C. & Hauert, C. Coevolutionary dynamics: From finite to infinite populations. *Phys. Rev. Lett.* **95**, 238701 (2005). 16384353 10.1103/PhysRevLett.95.238701

[52] Altrock, P. M. & Traulsen, A. Fixation times in evolutionary games under weak selection. *New J. Phys.* **11**, 013012 (2009).

[53] Tkadlec, J., Pavlogiannis, A., Chatterjee, K. & Nowak, M. A. Population structure determines the tradeoff between fixation probability and fixation time. *Commun. Biol.* **2**, 138 (2019). 31044163 10.1038/s42003-019-0373-y PMC6478818

[54] Fisher, R. A. *The Genetical Theory of Natural Selection* (Clarendon Press, 1930).

[55] Taylor, P. D. Allele-Frequency Change in a Class-Structured Population. *Am. Nat.* **135**, 95–106 (1990).

[56] Cox, J. T. Coalescing random walks and voter model consensus times on the torus in **Z**^d^. *Ann. Probab.* **17**, 1333–1366 (1989).

[57] Li, A., Meng, Y., Zhou, L., Masuda, N. & Wang, L. Temporality modulates the effect of network heterogeneity on cooperation fixation. GitHub 10.5281/zenodo.18114616 (2026). 10.1038/s41467-026-72717-w PMC13369883 42103702

