# Supplementary material for: Temporality modulates the effect of network heterogeneity on cooperation fixation
Source: Nat Commun. 2026 May 8;17:6238. doi: 10.1038/s41467-026-72717-w (PMC13369883; doi:10.1038/s41467-026-72717-w)
Supplement: Supplementary file 1 — Supplementary Information [file 41467_2026_72717_MOESM1_ESM.pdf]

Supplementary Information for

Temporality modulates the effect of network heterogeneity on cooperation fixation

Aming Li, Yao Meng, Lei Zhou, Naoki Masuda & Long Wang

Supplementary Note 1: Reproductive-value-weighted frequency of cooperators

Here we derive the reproductive-value-weighted frequency of cooperators through generations in the limit of weak selection, which is presented by Eq. (8) in the main text. Population structure is represented by a connected and undirected graph G with N individuals, and edge weight $w_{ij} = w_{ji}$ represents the intensity of interactions and $w_{ii} = 0$. The weighted degree of node i is defined by $w_i = \sum_{j=1}^{N} w_{ji}$, and $W = \sum_{i=1}^{N} w_i$ represents the total weight of G. The probability of a random walk from node i to node j in a single step is denoted by $p_{ij} = w_{ij}/w_i$.

The state of the evolutionary process is given by $\mathbf{x} \in \{0,1\}^N$, where $x_i = 1$ indicates that the individual i cooperates, and $x_i = 0$ indicates that i defects. The evolutionary process on static networks is equivalent to a discrete-time Markov chain $\mathbf{X}(t)$ on $\{0,1\}^N$. At each time step t, an individual i is randomly selected to update its strategy. Then, individual i imitates the strategy of neighbor j with probability proportional to $w_{ij}F_j(\mathbf{x})$ or remains in its current strategy with probability proportional to $F_i(\mathbf{x})$, where $F_j(\mathbf{x})$ is the fitness of individual j in state $\mathbf{x}$. We define the replacement graph $\tilde{w}_{ij}$ capturing who imitates the strategy from whom, where $\tilde{w}_{ii} = 1$ and $\tilde{w}_{ij} = w_{ij}$ if $i \neq j$. The probability of a random walk from node i to j on this replacement graph in a single step is denoted by $\tilde{p}_{ij} = \tilde{w}_{ij}/\tilde{w}_i$, where $\tilde{w}_i = w_i + 1$, and the probability of a n-step random walk from node i to j is denoted by $\tilde{p}_{ij}^{(n)}$.

The probability that i copies j's strategy in state $\mathbf{x}$ is

$$e_{ji}(\mathbf{x}) = \frac{1}{N}\frac{\tilde{w}_{ij}F_j(\mathbf{x})}{\sum_{l=1}^{N}\tilde{w}_{il}F_l(\mathbf{x})}. \tag{1}$$

The payoff matrix for the donation games is

$$\begin{array}{c} \\ \mathrm{C} \\ \mathrm{D} \end{array}\!\!\begin{array}{c} \begin{array}{cc} \mathrm{C} & \mathrm{D} \end{array} \\ \begin{pmatrix} b-c & -c \\ b & 0 \end{pmatrix} \end{array}.$$

Then, the accumulated weighted payoff for individual i in state $\mathbf{x}$ is

$$\begin{aligned} f_i(\mathbf{x}) &= \sum_{l=1}^{N} w_{il}\left[(b-c)x_i x_l - c x_i(1-x_l) + b(1-x_i)x_l\right] \\ &= w_i\left(-c x_i + b\sum_{l=1}^{N} p_{il}x_l\right). \end{aligned}$$

We first calculate the reproductive value $\tilde{\pi}_i$ of vertex i [1, 2], which is the unique solution of equations

$$\begin{aligned} \sum_{j=1}^{N} e^{\circ}_{ij}\tilde{\pi}_j &= \sum_{j=1}^{N} e^{\circ}_{ji}\tilde{\pi}_i, \\ \sum_{i=1}^{N} \tilde{\pi}_i &= 1, \end{aligned} \tag{2}$$

where $e^{\circ}_{ji} = \tilde{p}_{ij}/N$ defines the probability of j replacing i under neutral drift ($\delta = 0$), and $F_i(\mathbf{x}) = 1$ for any i. Here we employ the superscript $^{\circ}$ to represent the quantities under neutral drift. According to Eq. (1), we know that $e^{\circ}_{ij} = \tilde{p}_{ji}/N$. Then, the reproductive value $\tilde{\pi}_i$ is given by

$$\tilde{\pi}_i = \frac{w_i + 1}{W + N}.$$

We define the reproductive-value-weighted frequency of cooperators in state $\mathbf{x}$ by

$$\hat{x} = \sum_{i=1}^{N} \tilde{\pi}_i x_i.$$

The expected reproductive-value-weighted frequency of cooperators at time step $g+1$ starting from the initial state $\boldsymbol{\xi} \in \{0,1\}^N$ is given by

$$\begin{aligned} \mathbb{E}_{\boldsymbol{\xi}}[\hat{x}(g+1)] &= \mathbb{E}_{\boldsymbol{\xi}}\left[\hat{x}(0)\right] + \sum_{t=0}^{g}\left(\mathbb{E}_{\boldsymbol{\xi}}\left[\hat{x}(t+1)\right] - \mathbb{E}_{\boldsymbol{\xi}}\left[\hat{x}(t)\right]\right) \\ &= \mathbb{E}_{\boldsymbol{\xi}}\left[\hat{x}(0)\right] + \sum_{t=0}^{g}\sum_{\mathbf{x}} \mathbb{P}_{\boldsymbol{\xi}}\left[\mathbf{X}(t) = \mathbf{x}\right]\mathbb{E}\left[\hat{x}(t+1) - \hat{x}(t) | \mathbf{X}(t) = \mathbf{x}\right], \end{aligned}$$

where $\mathbb{P}_{\boldsymbol{\xi}}\big[\mathbf{X}(t) = \mathbf{x}\big]$ represents the probability that the evolutionary process starting from state $\boldsymbol{\xi}$ reaches state $\mathbf{x}$ at time step t. We now define function $D(\mathbf{x}) := \mathbb{E}\big[\hat{x}(t+1) - \hat{x}(t) | \mathbf{X}(t) = \mathbf{x}\big]$ as the expected change of $\hat{x}$ from state $\mathbf{x}$. One obtains [1, 2]

$$D(\mathbf{x}) = \sum_{i=1}^{N} \tilde{\pi}_i \sum_{j=1}^{N} (x_j - x_i)\, e_{ji}(\mathbf{x}). \tag{3}$$

Note that $D^{\circ}(\mathbf{x}) = 0$ for any state $\mathbf{x} \in \{0,1\}^N$ according to Eq. (2).

By considering the asymptotic expansion of $\mathbb{E}_{\boldsymbol{\xi}}[\hat{x}(g+1)]$ as $\delta \to 0^+$, we obtain

$$\begin{aligned}
\mathbb{E}_{\boldsymbol{\xi}}[\hat{x}(g+1)] &= \mathbb{E}_{\boldsymbol{\xi}}\left[\hat{x}(0)\right] + \sum_{t=0}^{g} \sum_{\mathbf{x}} \mathbb{P}_{\boldsymbol{\xi}}\big[\mathbf{X}(t) = \mathbf{x}\big] D(\mathbf{x}) \\
&= \mathbb{E}_{\boldsymbol{\xi}}\left[\hat{x}(0)\right] + \delta \sum_{t=0}^{g} \sum_{\mathbf{x}} \mathbb{P}^{\circ}_{\boldsymbol{\xi}}\big[\mathbf{X}(t) = \mathbf{x}\big] \left.\frac{\mathrm{d}D(\mathbf{x})}{\mathrm{d}\delta}\right|_{\delta=0} + \mathcal{O}\left(\delta^2\right) \\
&= \mathbb{E}_{\boldsymbol{\xi}}\left[\hat{x}(0)\right] + \delta \sum_{t=0}^{g} \mathbb{E}^{\circ}_{\boldsymbol{\xi}}\left[\left.\frac{\mathrm{d}D\left(\mathbf{X}(t)\right)}{\mathrm{d}\delta}\right|_{\delta=0}\right] + \mathcal{O}\left(\delta^2\right).
\end{aligned}$$

For the initial state of l cooperators uniformly randomly located on the graph, and the expected reproductive-value-weighted frequency of cooperators is given by

$$\mathbb{E}_{\mathbf{u}}[\hat{x}(g+1)] = \frac{l}{N} + \delta \sum_{t=0}^{g} \mathbb{E}^{\circ}_{\mathbf{u}}\left[\left.\frac{\mathrm{d}D\left(\mathbf{X}(t)\right)}{\mathrm{d}\delta}\right|_{\delta=0}\right] + \mathcal{O}\left(\delta^2\right), \tag{4}$$

where the subscript $\mathbf{u}$ represents that the initial l cooperators are randomly placed on the graph with the uniform probability. Using Eq. (3), we obtain

$$\left.\frac{\mathrm{d}D(\mathbf{x})}{\mathrm{d}\delta}\right|_{\delta=0} = \sum_{i=1}^{N} \tilde{\pi}_i \sum_{j=1}^{N} (x_j - x_i) \left.\frac{\mathrm{d}e_{ji}(\mathbf{x})}{\mathrm{d}\delta}\right|_{\delta=0},$$

where

$$\left.\frac{\mathrm{d}e_{ji}(\mathbf{x})}{\mathrm{d}\delta}\right|_{\delta=0} = \frac{\tilde{p}_{ij}}{N}(f_j(\mathbf{x}) - \sum_{l=1}^{N} \tilde{p}_{il} f_l(\mathbf{x})).$$

Thus, $\left.\frac{\mathrm{d}D(\mathbf{x})}{\mathrm{d}\delta}\right|_{\delta=0}$ is given by

$$
\begin{aligned}
\left.\frac{\mathrm{d}D(\mathbf{x})}{\mathrm{d}\delta}\right|_{\delta=0} &= \frac{1}{N}\sum_{i,j=1}^{N}\tilde{\pi}_i(x_j-x_i)\tilde{p}_{ij}\left(f_j(\mathbf{x})-\sum_{l=1}^{N}\tilde{p}_{il}f_l(\mathbf{x})\right)\\
&= -\frac{c}{N}\left(\sum_{i=1}^{N}\tilde{\pi}_i w_i x_i^2-\sum_{i,j=1}^{N}\tilde{\pi}_i\tilde{p}_{ij}^{(2)}w_jx_ix_j\right)\\
&+\frac{b}{N}\left(\sum_{i,j=1}^{N}\tilde{\pi}_iw_ip_{ij}x_ix_j-\sum_{i,j,k=1}^{N}\tilde{\pi}_i\tilde{p}_{ij}^{(2)}w_jp_{jk}x_ix_k\right)
\end{aligned}
\tag{5}
$$

By operating $\sum_{t=0}^{g}\mathbb{E}_{\mathbf{u}}^{\circ}[\cdot]$ on both sides of Eq. (5), the calculation of $\sum_{t=0}^{g}\mathbb{E}_{\mathbf{u}}^{\circ}\left[\left.\frac{\mathrm{d}D(\mathbf{X}(t))}{\mathrm{d}\delta}\right|_{\delta=0}\right]$ in Eq. (4) is to compute the linear combination of $\sum_{t=0}^{g}\mathbb{E}_{\mathbf{u}}^{\circ}\left[x_i(t)x_j(t)\right]$ on the right side of Eq. (5).

According to the coalescent theory [3], we have

$$
\begin{aligned}
\mathbb{E}_{\mathbf{u}}^{\circ}\left[x_i(t)x_j(t)\right] &= \frac{l}{N}\mathbb{P}_{(i,j)}^{\mathrm{CRW}}\left[T_{\mathrm{coal}}\leq t\right]+\frac{l(l-1)}{N(N-1)}\left(1-\mathbb{P}_{(i,j)}^{\mathrm{CRW}}\left[T_{\mathrm{coal}}\leq t\right]\right)\\
&= \frac{l}{N}\left(1-\frac{N-l}{N-1}\mathbb{P}_{(i,j)}^{\mathrm{CRW}}\left[T_{\mathrm{coal}}>t\right]\right),
\end{aligned}
$$

$\mathbb{P}_{(i,j)}^{\mathrm{CRW}}\left[T_{\mathrm{coal}}=n\right]$ represents the probability that two random walkers starting from node i and node j coalescent at time step n. At each time step during the evolutionary process, each of the two random walkers is chosen to take a step of random walk with probability $1/N$. We now only consider two independent random walks starting from i and j on the replacement network with coalescence time τ, where each of the random walkers is chosen with equal probability $1/2$ at each time step. Therefore, the coalescence time of two random workers in the evolutionary process can be approximately rescaled by a factor of $2/N$. Let $\mathrm{P}_{\{i,j\}}(\tau)$ denote the probability that two random walkers starting at node i and j coalescence at time τ. When $\tau>0$, we obtain

$$
\mathrm{P}_{\{i,j\}}(\tau)=\begin{cases}\frac{1}{2}\sum_{k=1}^{N}\tilde{p}_{ik}\mathrm{P}_{\{k,j\}}(\tau-1)+\frac{1}{2}\sum_{k=1}^{N}\tilde{p}_{jk}\mathrm{P}_{\{k,i\}}(\tau-1), & \text{if } i\neq j\\ 0, & \text{if } i=j.\end{cases}
$$

And the probability of coalescing at time 0 is

$$
\mathrm{P}_{\{i,j\}}(0)=\begin{cases}0 & \text{if } i\neq j\\ 1 & \text{if } i=j.\end{cases}
\tag{6}
$$

Therefore, we have

$$\sum_{t=0}^{g} \mathbb{E}_{\mathbf{u}}^{\circ}\left[\frac{l}{N} - x_i(t)x_j(t)\right] = \frac{l(N-l)}{N(N-1)} \sum_{t=0}^{g} \mathbb{P}_{(i,j)}^{\mathrm{CRW}}\left[T_{\mathrm{coal}} > t\right]$$
$$= \frac{l(N-l)}{2(N-1)} \sum_{t=0}^{\lfloor 2g/N \rfloor} \sum_{\tau=t+1}^{\infty} \mathrm{P}_{\{i,j\}}(\tau).$$

By using $\tilde{\tau}_{ij}\left(T\right) = \sum_{t=0}^{T} \sum_{\tau=t+1}^{\infty} \mathrm{P}_{\{i,j\}}(\tau)$, we have $\mathbb{E}_{\mathbf{u}}[\hat{x}(g)]$ starting from l cooperators as follows:

$$\mathbb{E}_{\mathbf{u}}[\hat{x}(g)] = \frac{l}{N} + \frac{l(N-l)\delta}{2N(N-1)}\left[-c\sum_{i,j} \tilde{\pi}_i \tilde{p}_{ij}^{(2)} w_j \tilde{\tau}_{ij}\left(T\right) \right.$$
$$\left. + b\left(\sum_{i,j,k} \tilde{\pi}_i \tilde{p}_{ij}^{(2)} w_j p_{jk} \tilde{\tau}_{ik}\left(T\right) - \sum_{i,j} \tilde{\pi}_i w_i p_{ij} \tilde{\tau}_{ij}\left(T\right)\right)\right],$$

where $T = \lfloor 2(g-1)/N \rfloor$. As g tends to infinity, the fixation probability of cooperation is given by

$$\rho_C = \frac{1}{N} + \frac{\delta}{2N}\left[-c\sum_{i,j} \tilde{\pi}_i \tilde{p}_{ij}^{(2)} w_j \tau_{ij} + b\left(\sum_{i,j,k} \tilde{\pi}_i \tilde{p}_{ij}^{(2)} w_j p_{jk} \tau_{ik} - \sum_{i,j} \tilde{\pi}_i w_i p_{ij} \tau_{ij}\right)\right], \quad (7)$$

where τ_{ij} is the expected coalescence time from i and j, which is calculated by

$$\tau_{ij} = \sum_{\tau=0}^{\infty} \tau \mathrm{P}_{\{i,j\}}(\tau). \quad (8)$$

Note that τ_{ij} can also be obtained by solving the recurrence relation

$$\tau_{ij} = \begin{cases} 1 + \frac{1}{2}\sum_{k=1}^{N} \tilde{p}_{ik}\tau_{kj} + \frac{1}{2}\sum_{k=1}^{N} \tilde{p}_{jk}\tau_{ki}, & \text{if } i \neq j \\ 0, & \text{if } i = j. \end{cases} \quad (9)$$

Supplementary Note 2: Critical benefit-to-cost ratio

We next derive the critical benefit-to-cost ratio on static unweighted graphs. According to Eq. (7), the critical benefit-to-cost ratio is

$$(b/c)^* = \frac{\sum_{i,j} \tilde{\pi}_i \tilde{p}_{ij}^{(2)} w_j \tau_{ij}}{\sum_{i,j,k} \tilde{\pi}_i \tilde{p}_{ij}^{(2)} w_j p_{jk} \tau_{ik} - \sum_{i,j} \tilde{\pi}_i w_i p_{ij} \tau_{ij}}.$$

By defining $\tau^{(n)} = \sum_{i,j=1}^{N} \tilde{\pi}_i \tilde{p}_{ij}^{(n)} \tau_{ij}$ and combining the recurrence relation given in Eq. (9), we have

$$\tau^{(n+1)} = \tau^{(n)} + \sum_{i=1}^{N} \tilde{\pi}_i \tilde{p}_{ii}^{(n)} \tau_{ii}^{+} - 1, \tag{10}$$

where $\tau_{ii}^{+} = 1 + \sum_{j=1}^{N} \tilde{p}_{ij} \tau_{ij}$. For $n = 1, 2$, and 3, we have

$$\begin{aligned}
\tau^{(1)} &= \sum_{i=1}^{N} \tilde{\pi}_i \tau_{ii}^{+} - 1, \\
\tau^{(2)} &= \sum_{i=1}^{N} \tilde{\pi}_i \tau_{ii}^{+} \left(1 + \tilde{p}_{ii}^{(1)}\right) - 2, \\
\tau^{(3)} &= \sum_{i=1}^{N} \tilde{\pi}_i \tau_{ii}^{+} \left(1 + \tilde{p}_{ii}^{(1)} + \tilde{p}_{ii}^{(2)}\right) - 3,
\end{aligned}$$

where $\tilde{p}_{ii}^{(1)} = 1/(w_i + 1)$. By letting $n \to \infty$ in Eq. (10), we further obtain

$$\sum_{i=1}^{N} \tilde{\pi}_i^2 \tau_{ii}^{+} = 1. \tag{11}$$

By replacing the weighted degree w_i in Eq. (7) with the average degree k, we have the critical ratio

$$(b/c)^* \approx \frac{\frac{k}{k+1} \tau^{(2)}}{\tau^{(3)} - \tau^{(1)} - \frac{1}{k+1} \tau^{(2)}}.$$

We represent τ_{ii}^{+} in Eq. (11) by the mean value

$$\tau_{ii}^{+} \approx \frac{N(k+1)^2}{\langle k^2 \rangle + 2k + 1}, \tag{12}$$

where $\langle k^2 \rangle$ is the second moment of the degree distribution. With the equation $\sum_i \tilde{\pi}_i p_{ii}^{(2)} = 1/(k+1)$, we obtain the critical ratio as follows:

$$\begin{aligned}
(b/c)^* &\approx \frac{\tau_{ii}^{+}(k+2)/(k+1) - 2}{\tau_{ii}^{+}/(k+1) - 2} \\
&= \frac{N(k+1)(k+2) - 2(\langle k^2 \rangle + 2k + 1)}{N(k+1) - 2(\langle k^2 \rangle + 2k + 1)} \\
&= \frac{N\,(k+2) - 2\,(k+1)}{N - 2\,(k+1)} + \frac{2}{\left(1 - \frac{2k+2}{N}\right)^2} \frac{\mathrm{var}(k)}{N} + \mathcal{O}\left(\frac{\mathrm{var}(k)}{N}\right)^2,
\end{aligned} \tag{13}$$

where var(k) is the variance of the degree distribution. Equation (13) indicates that the critical ratio for heterogeneous networks (i.e., networks in which different nodes may have different degrees) is larger than that for regular networks (i.e., those in which all nodes have the same degree). For regular networks, the right-hand side of Eq. (13) becomes $k+2$ in the limit $N \to \infty$. In this manner, we recover the $b/c > k+2$ rule for the imitation updating on large regular graphs [4].

Supplementary Note 3: Description of empirical datasets

Temporal networks are constructed from four empirical datasets. All the datasets are provided by the SocioPatterns collaboration [5]. The contact time duration is measured as a multiple of 20 seconds, where each contact has the form "t, i, j", indicating person i and person j have a proximity relationship at interval $[t - 20\mathrm{s}, \mathrm{t})$.

The ACM Conference dataset was collected during the ACM Hypertext 2009 conference and from 113 attendees of the conference over about 2.5 days starting from 8am on Jun 29, 2009 [6]. The Student dataset represents the contacts among 180 students during seven days in five classes in a high school in Marseilles, France in 2012 [7]. The Office dataset represents the contacts among 92 individuals in an office building in France, recorded from June 24 to July 3, 2013 [8]. The Hospital ward dataset represents the contacts among 75 individuals in a hospital ward in Lyon, France, from December 6 to December 10 in 2010 [9]. For more details, please see Table 1 and Supplementary Figs. 10–12.

Supplementary Table 1: Statistics of the empirical datasets. Variable N is the number of nodes; Δt is the duration of each snapshot, where a valid snapshot is one with at least one edge in the snapshot. We use two values of Δt for each dataset shown in the table, separated by a comma. M is the number of snapshots; $\langle k^2 \rangle$ denotes the average second moment of degree in snapshots of empirical temporal networks.

	ACM conference	Student 2012	Office 2013	Hospital 2013
N	113	180	92	75
Δt (hour)	0.5, 6	2, 12	2, 12	0.5, 6
M	81, 9	49, 13	58, 10	162, 17
$\langle k^2 \rangle$	3.83, 51.23	4.29, 16.17	1.43, 8.77	4.73, 36.28

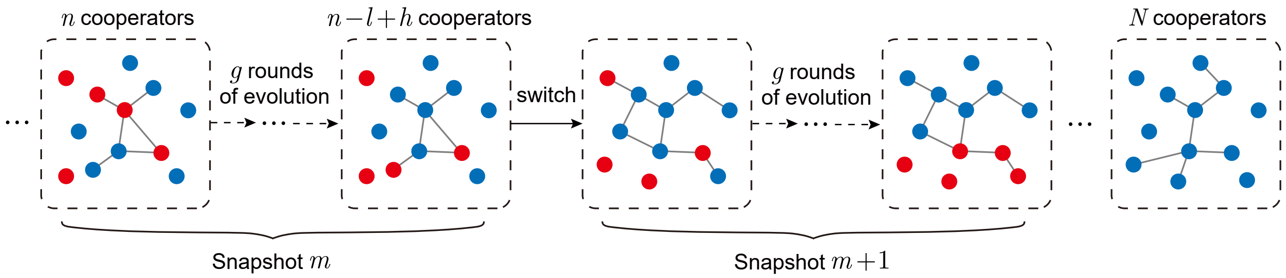

Supplementary Figure 1: Illustration of the evolutionary process on temporal networks. We describe the evolutionary game process of N players on temporal networks. At the beginning of the mth snapshot, n cooperators (blue circles) are randomly placed across the snapshot, including the largest connected component, while the remaining cooperators are placed on isolated nodes. Starting from l cooperators on the largest connected component, after g rounds of evolution, there are h cooperators on the largest connected component and $n - l$ cooperators on isolated nodes, giving a total of $n - l + h$ cooperators in the snapshot. Then, the temporal network switches to snapshot $m + 1$, and the evolutionary dynamics proceed for another g rounds. The evolutionary process ends when the network reaches the state of full cooperation or full defection.

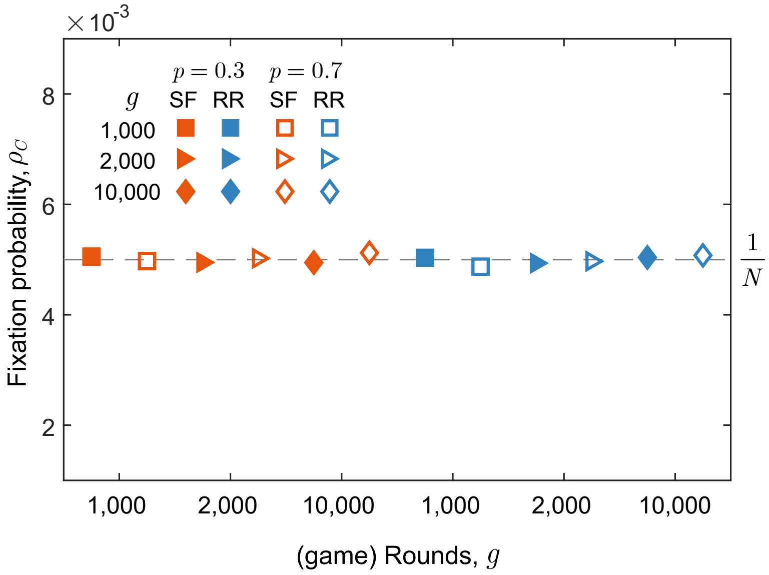

Supplementary Figure 2: Fixation probability on temporal networks under neutral drift. We numerically obtain the fixation probability on temporal scale-free (SF) and random regular (RR) networks by setting the intensity of selection $\delta = 0$. The obtained fixation probability is consistent with that in the static case $(1/N)$, where N is the total number of nodes in the network. This result holds for both temporal SF and RR networks across different evolutionary timescales g and edge densities p. We construct each snapshot of the synthetic temporal network by randomly choosing a fraction p of edges from the corresponding static networks ($p = 0.3, 0.7$). The other parameters are the same as those used in Fig. 2 in the main text.

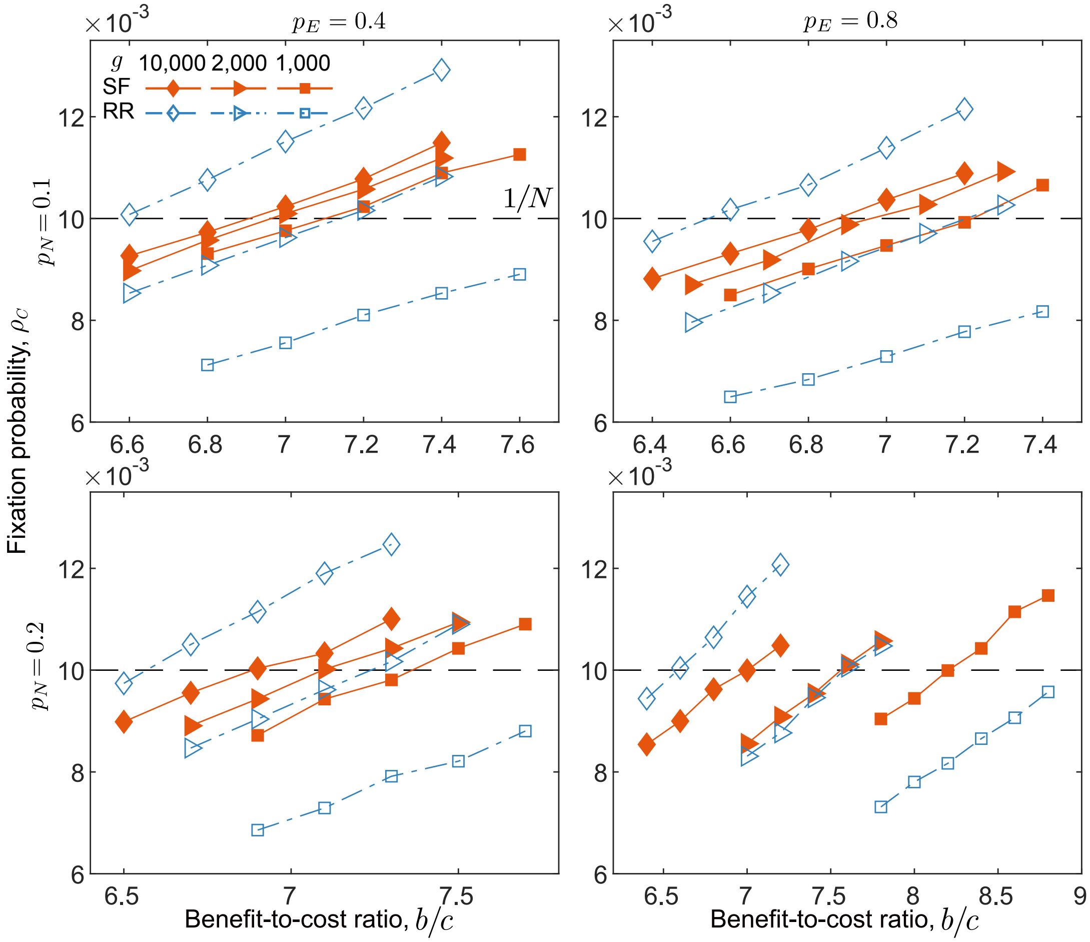

Supplementary Figure 3: Fixation of cooperation on temporal networks constructed by the rewiring model. We generate the heterogeneous temporal networks using the rewiring model starting from a scale-free network. We numerically calculate the fixation probability of cooperation across different network parameters ($p_N = 0.1, 0.2$; $p_E = 0.4, 0.8$) and evolutionary timescale $g = 1000, 2000, 10000$. As a comparison, we numerically calculate the fixation probability on the corresponding homogeneous counterparts. We show that, for all network parameter settings, when the evolutionary timescale g is relatively small, the heterogeneous temporal networks promote cooperation more than the homogeneous counterparts, despite that only a small proportion of nodes break and rewire. However, temporal homogeneous networks show a higher fixation probability at $g = 10000$. This result implies that sufficient evolutionary time allows cooperative clusters to form and persist even on homogeneous networks, thereby diminishing the advantage of heterogeneous networks with short fixation times.

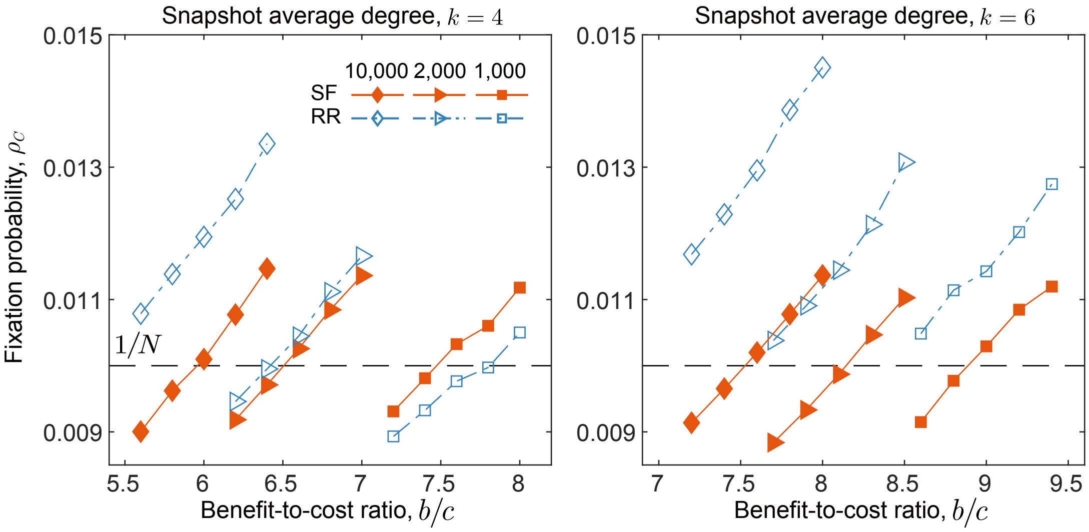

Supplementary Figure 4: Fixation of cooperation on temporal networks constructed by the activity-driven model. We generate the temporal networks using the activity-driven model with a power-law activity distribution with the exponent $\gamma = 2.5$. We numerically calculate the fixation probability of cooperation across different snapshot average degree ($k = 4, 6$) and evolutionary timescale $g = 1000, 2000, 10000$. As a comparison, we numerically calculate the fixation probability on the corresponding homogeneous counterparts. We show that, for sparse networks ($k = 4$), when the evolutionary timescale g is relatively small, the heterogeneous temporal networks promote cooperation more than their homogeneous counterparts. In contrast, for dense networks ($k = 6$), the homogeneous networks outperform the heterogeneous temporal networks in promoting the fixation of cooperation across the three values of g, which is consistent with our results on both synthetic and empirical networks (Figs. 2d and 3d in the main text).

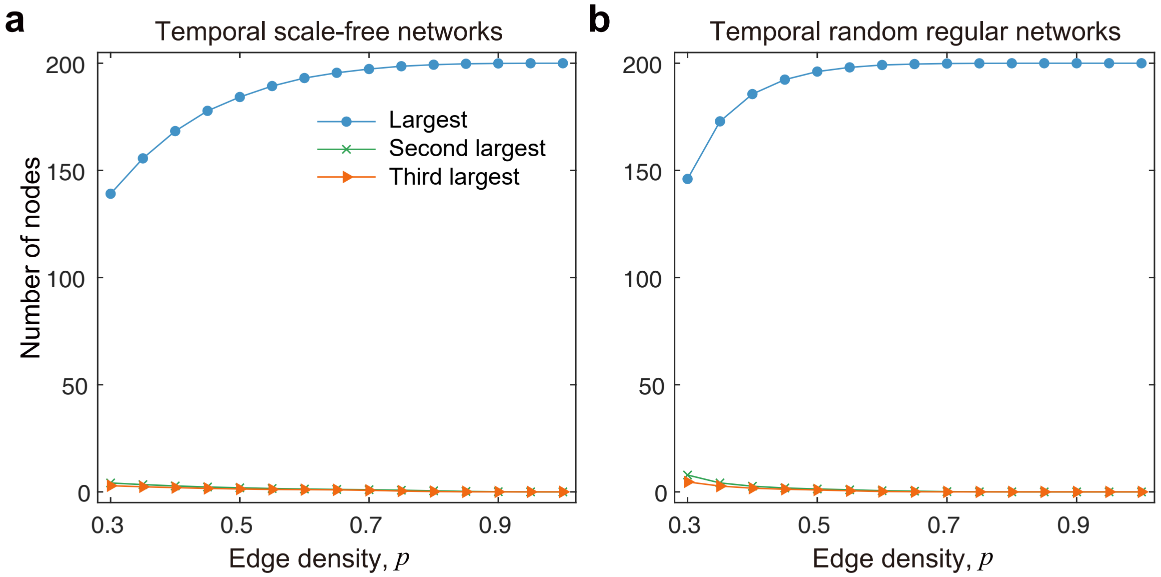

Supplementary Figure 5: Illustration on the number of nodes in components of the snapshot in different synthetic temporal scale-free and random regular networks. We show the average number of nodes in the three largest components in the snapshot of temporal scale-free networks (**a**) and random regular networks (**b**). As the fraction of active interactions p increases, the number of nodes in the largest connected component increases and approaches the network size. On the other hand, the second- and third-largest connected components contain only a small number of nodes compared to the largest connected component, and their sizes approach zero as p increases. Each marker represents the number of nodes averaged over 1000 snapshots. The synthetic temporal networks are generated with 200 nodes, and the aggregated static networks have an average degree of 6.

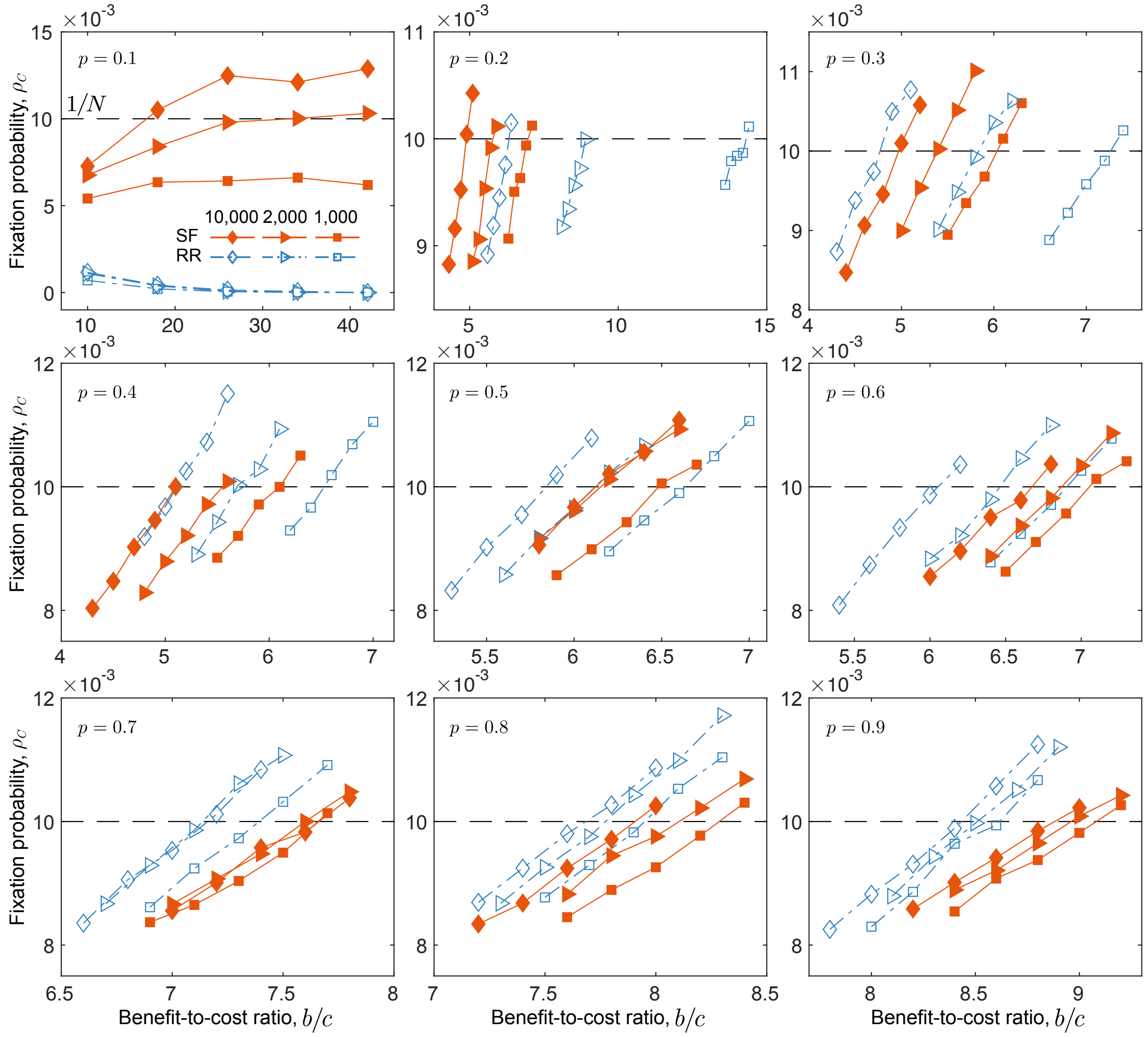

Supplementary Figure 6: Fixation of cooperation on synthetic temporal scale-free and random regular networks. We generate the synthetic temporal networks with edge density p from 0.1 to 0.9, and numerically calculate the fixation probability of cooperation across different evolutionary timescale $g = 1000, 2000, 10000$. The network size is $N = 100$. The other parameters are the same as those used in Fig. 2 in the main text.

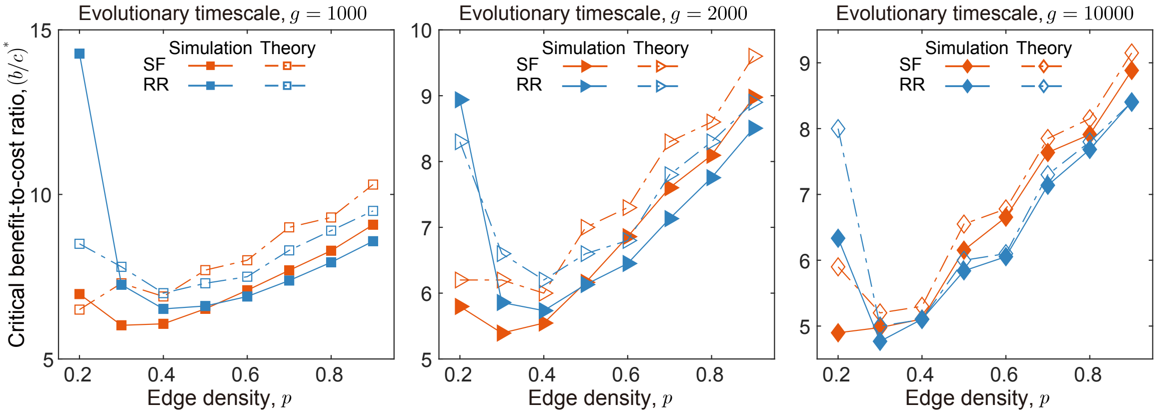

Supplementary Figure 7: Comparison of critical benefit-to-cost ratio between numerical and theoretical results. We theoretically calculate the critical benefit-to-cost ratio on synthetic temporal scale-free and random regular networks with edge density p ranging from 0.1 to 0.9, and compare the results with numerical simulations. We show that our theoretical calculations remain valid across different evolutionary timescales ($g = 1000, 2000, 10000$), with the discrepancy increasing when the networks are relatively sparse. In the case of a large evolutionary timescale g (e.g., $g = 10000$), our theoretical predictions present remarkable accuracy as edge density p increases.

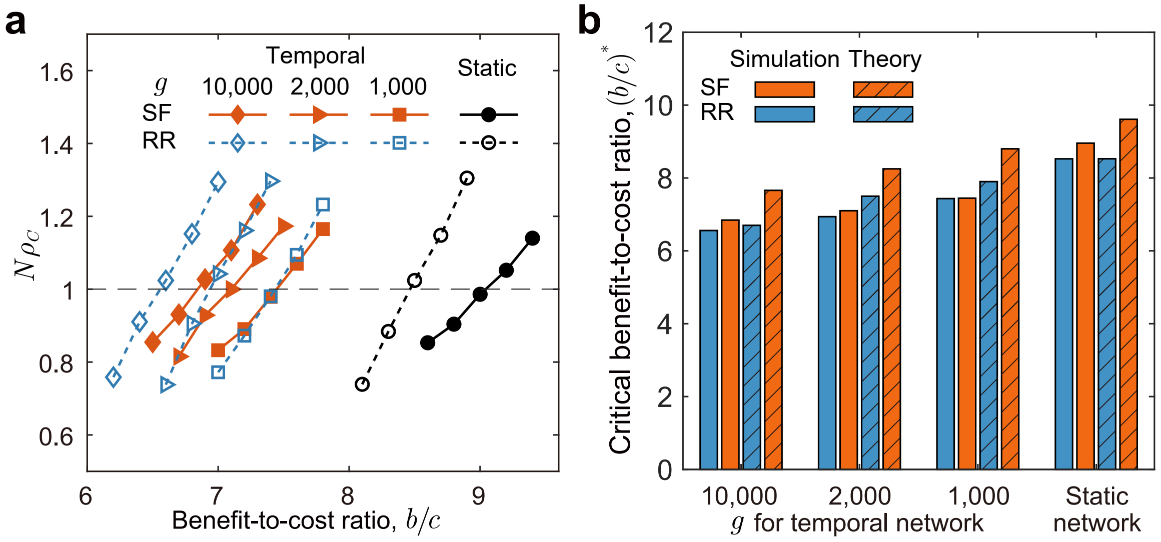

Supplementary Figure 8: Comparison of the critical benefit-to-cost ratio between synthetic temporal scale-free networks and random regular networks. **a**, Temporal random regular (RR) networks have a lower critical ratio $(b/c)^*$ than temporal scale-free (SF) networks over different values of evolutionary timescale g, which is consistent with the results for static networks. **b**, We further confirm the results shown in **a** with our theoretical calculations. Here each snapshot of the synthetic temporal network is constructed by randomly choosing a fraction of $p = 0.7$ edges from the corresponding static networks. The other parameters are the same as those in Fig. 2b.

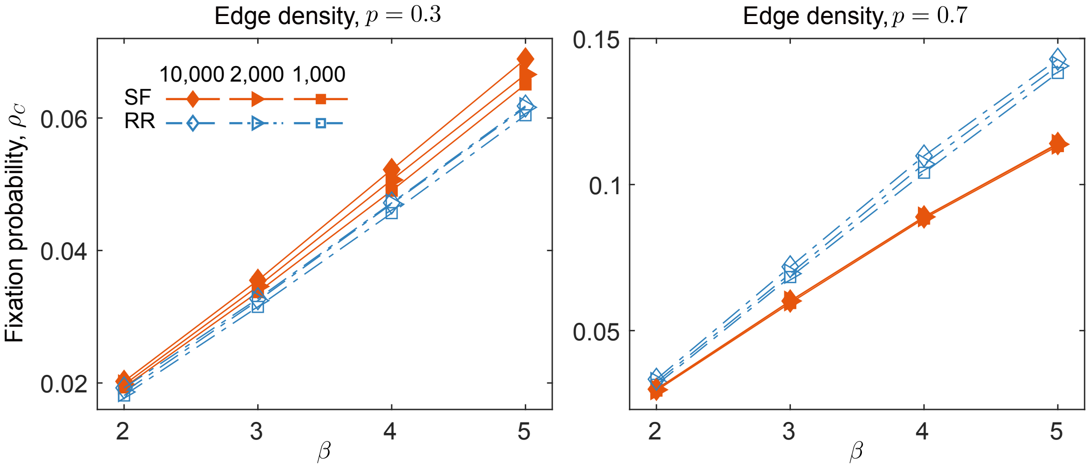

Supplementary Figure 9: Fixation of cooperation on temporal networks under the snowdrift game. We numerically calculate the fixation probability of cooperation on temporal scale-free networks across different edge densities ($p = 0.3, 0.7$) under a snowdrift game ($T = \beta, R = \beta - 1/2, S = \beta - 1, P = 0$). As a comparison, we numerically calculate the fixation probability on the corresponding homogeneous counterparts. We show that, when the edge density is low ($p = 0.3$), the heterogeneous temporal networks promote cooperation more than homogeneous counterparts across all evolutionary timescale g. However, the trend reverses for dense networks ($p = 0.7$). These results are consistent with our results on both synthetic and empirical networks (Figs. 2 and 3 in the main text). Here we generate the synthetic temporal networks with $N = 100$ and the average degree of the aggregated scale-free network is $k = 6$. The other parameters are the same as those used in Fig. 2 in the main text.

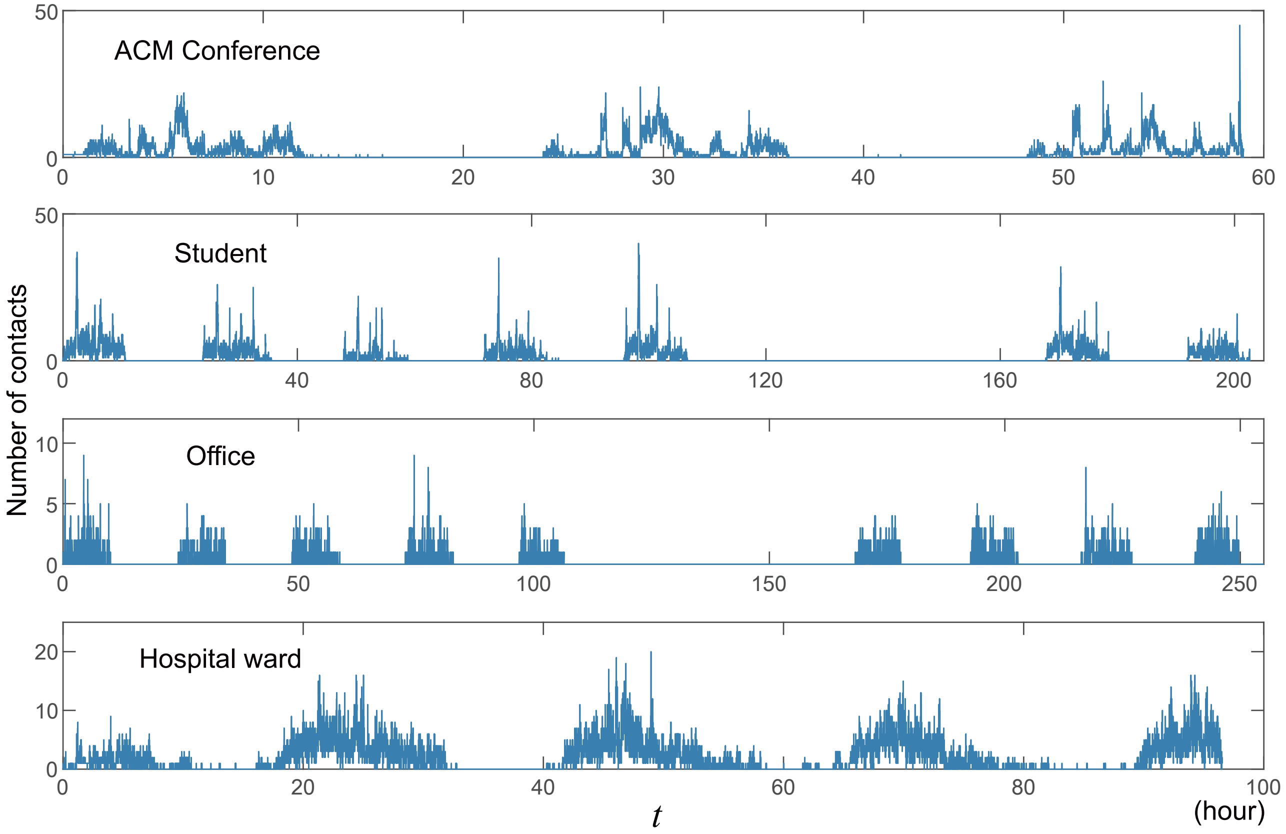

Supplementary Figure 10: Contact activity over time in empirical datasets. The curves show the number of contacts in each 20-second time window, which corresponds to the temporal resolution of the data. We observe circadian rhythms in all datasets, as well as days with no recorded contacts in the Student and Office datasets.

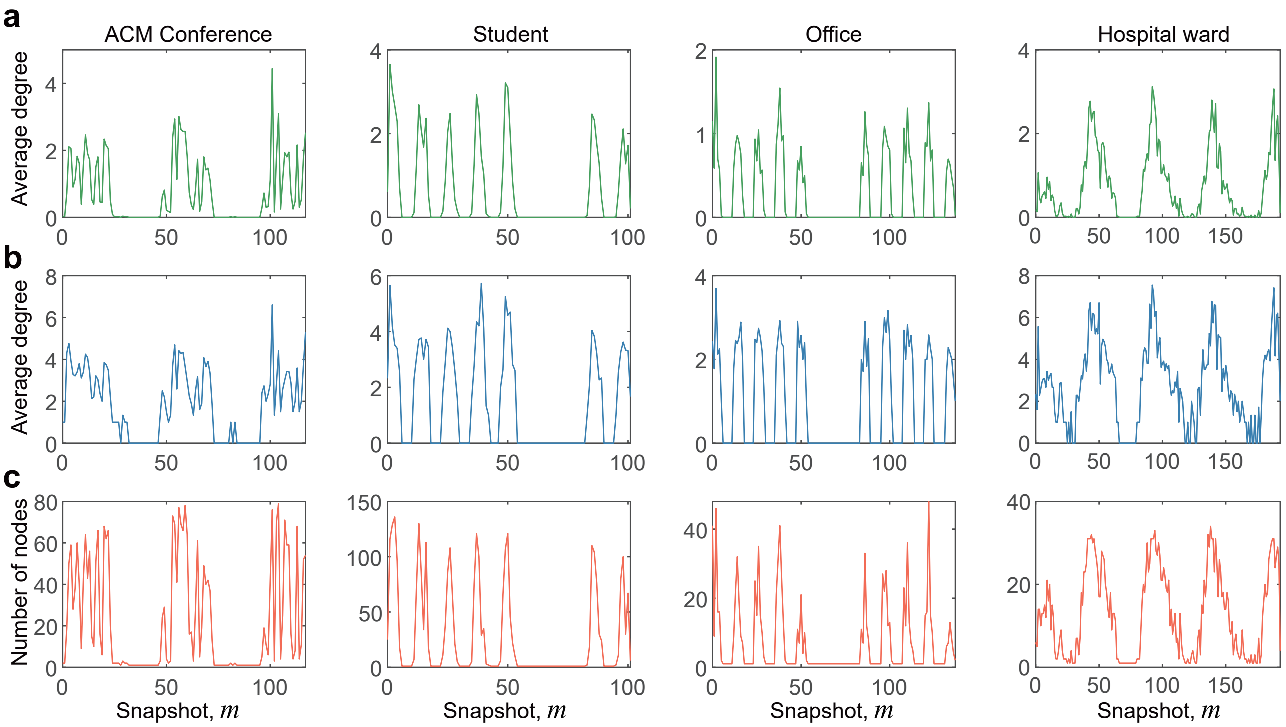

Supplementary Figure 11: Statistics across snapshots of empirical temporal networks. **a**, Average degree of each snapshot. **b**, Average degree of the largest connected component in each snapshot. **c**, Number of nodes in the largest connected component in each snapshot. The time windows for constructing temporal networks are the same as those used in Fig. 3c in the main text.

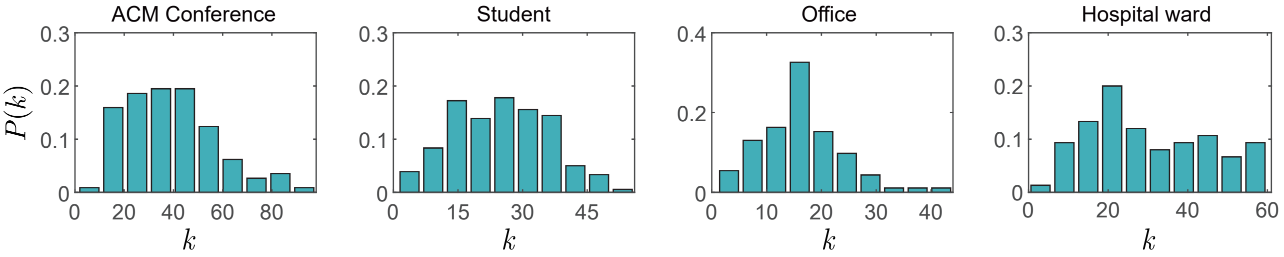

Supplementary Figure 12: Degree distribution of the aggregated static networks constructed from empirical datasets. We obtain the static networks by aggregating all contacts in the datasets over time. $P(k)$ indicates the fraction of nodes in each degree interval.

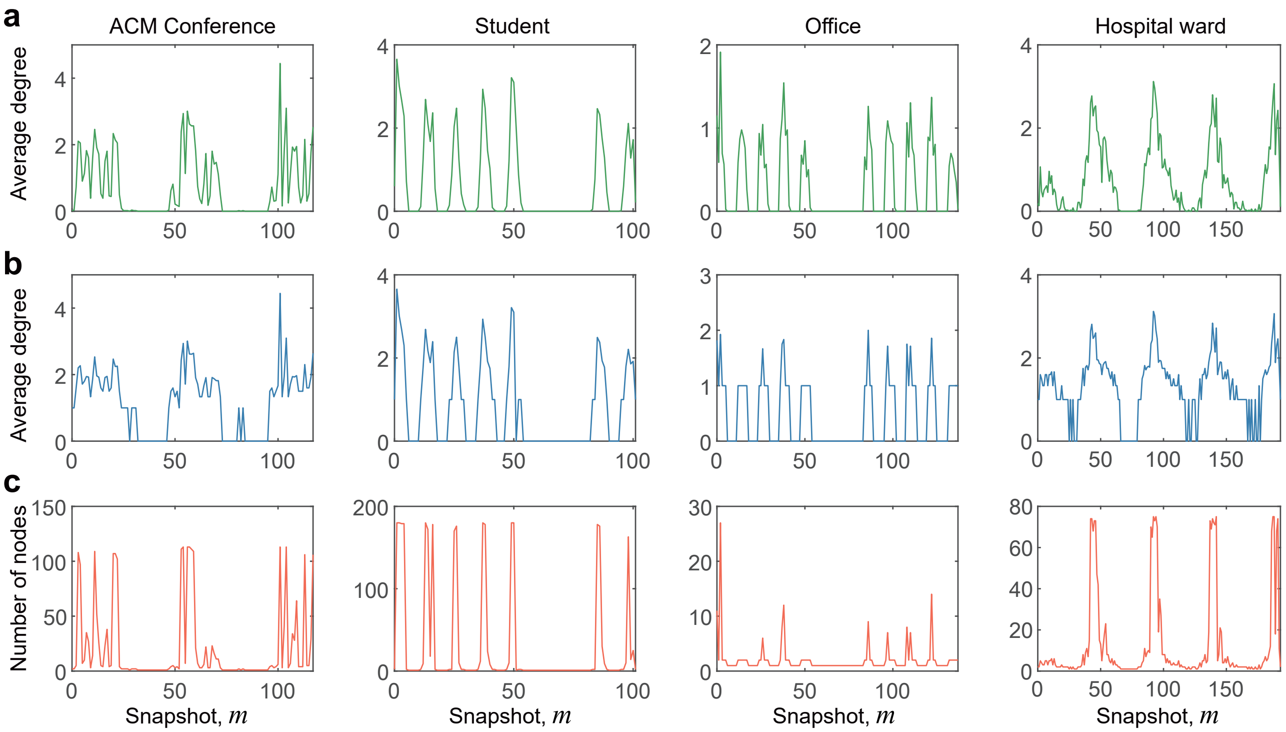

Supplementary Figure 13: Statistics across snapshots of the corresponding homogeneous temporal networks. **a**, Average degree of each snapshot in the temporal homogeneous networks constructed from each empirical dataset. **b**, Average degree of the largest connected component in each snapshot. **c**, Number of nodes in the largest connected component in each snapshot. Snapshots in synthetic temporal homogeneous networks are generated with the same number of edges as the corresponding snapshot in the empirical temporal networks. (Fig. 3 in the main text, Supplementary Fig. 11).

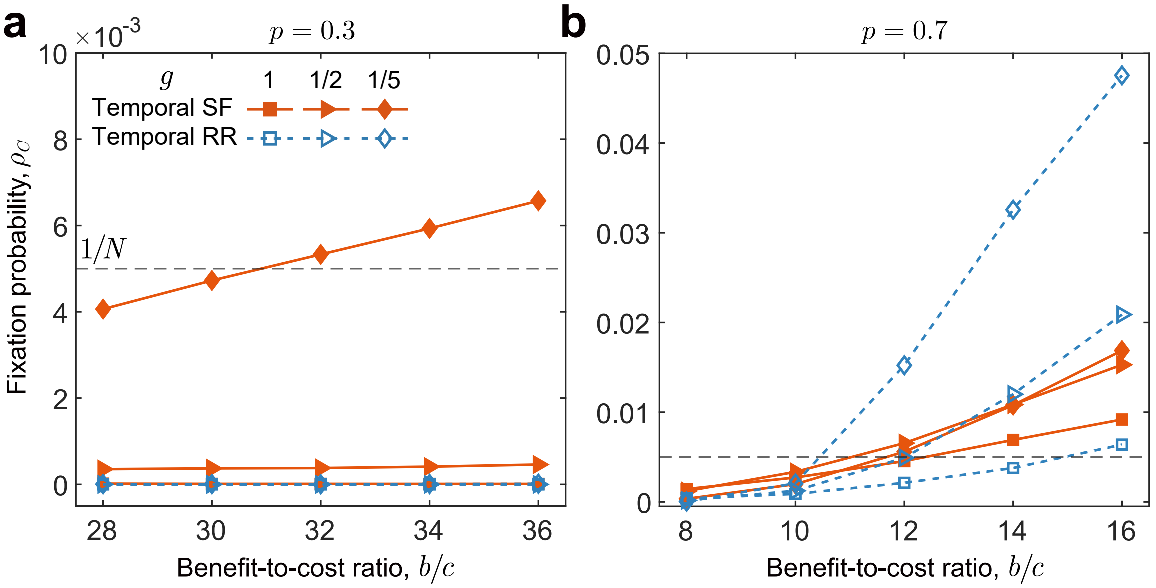

Supplementary Figure 14: Fixation probability of cooperation on rapidly changing temporal networks. **a**, For $g = 1, 1/2$ and $1/5$, where individuals update their strategies after interacting over $1/g$ snapshots, temporal scale-free networks ($p = 0.3$) exhibit a higher fixation probability than their random regular counterparts. **b**, When networks become dense ($p = 0.7$), temporal random regular networks are more effective at promoting the emergence of cooperation. Here we use the same network parameters as those used in Fig. 2a.

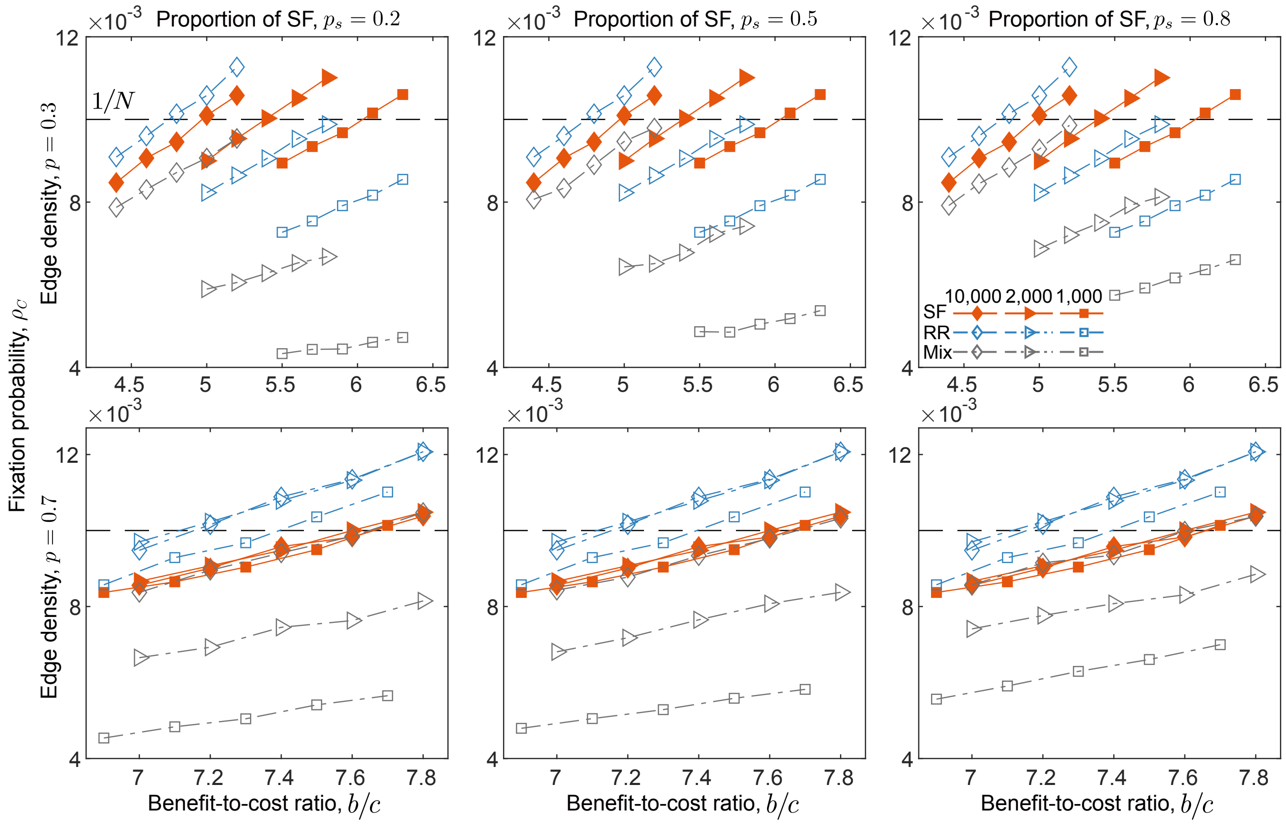

Supplementary Figure 15: Fixation of cooperation on temporal networks with alternate appearance of scale-free and random regular networks. We construct the mixed temporal network in which a fraction p_s of each evolutionary period g uses scale-free (SF) networks and the remaining fraction $1 - p_s$ uses random regular (RR) networks, with the network types alternating in successive periods. We numerically calculate the fixation probability of cooperation on the mixed temporal networks (markers in grey) across different edge densities ($p = 0.3$ in the first row, $p = 0.7$ in the second row) and proportions of SF subnetworks ($p = 0.2, 0.5, 0.8$ in columns from left to right). Then, we compare the results with those on the temporal SF (markers in orange) and RR (markers in blue) networks. We show that the mixed temporal networks present the lowest fixation probability in all parameter settings, with the least ability to promote cooperation. Here we generate the synthetic temporal networks with $N = 100$ and the average degree $k = 6$ for the aggregated scale-free networks. The other parameters are the same as those used in Fig. 2 in the main text.

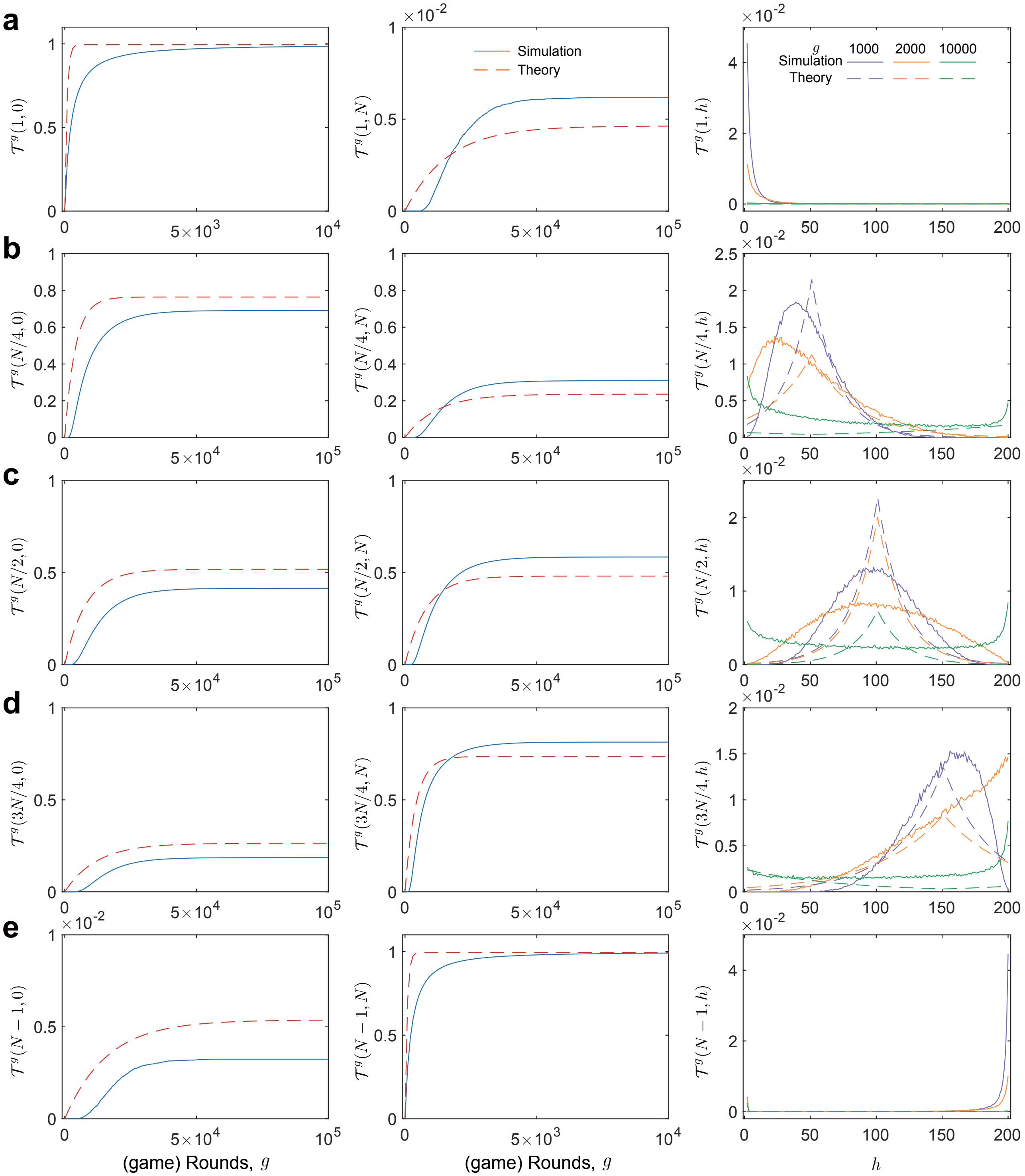

Supplementary Figure 16: The transition probability $\mathcal{T}^g(l,h)$ that the network starts from l cooperators and ends with h cooperators after g rounds of evolution. We numerically calculate the $\mathcal{T}^g(l,h)$ with $l = 1, N/4, N/2, 3N/4, N-1$ on a static scale-free network in **a-e**, respectively. The transition probability of reaching the fixation of cooperators or defectors over g rounds of evolution can be approximated using Eq. (11) in the main text (dashed line). The probability of reaching h cooperators ($0 < h < N$) at different values of g ($g = 1,000, 2,000, 10,000$) is shown in the right column. We set $N = 200$ and numerically calculate $\mathcal{T}^g(l,h)$ as the fraction of 2×10^5 runs that reach a state of h cooperators at round g, starting from l cooperators initially.

Supplementary References

[1] Allen, B. & McAvoy, A. A mathematical formalism for natural selection with arbitrary spatial and genetic structure. *J. Math. Biol.* **78**, 1147–1210 (2019).

[2] McAvoy, A. & Allen, B. Fixation probabilities in evolutionary dynamics under weak selection. *J. Math. Biol.* **82**, 1–41 (2021).

[3] Cox, J. T. Coalescing random walks and voter model consensus times on the torus in $\mathbf{Z}^{\mathrm{d}}$. *Ann. Probab.* **17**, 1333–1366 (1989).

[4] Ohtsuki, H., Hauert, C., Lieberman, E. & Nowak, M. A simple rule for the evolution of cooperation on graphs and social networks. *Nature* **441**, 502–505 (2006).

[5] Sociopatterns. `http://www.sociopatterns.org/`.

[6] Isella, L., Stehlé, J., Barrat, A., Cattuto, C., Pinton, J.-F. & Van den Broeck, W. What's in a crowd? analysis of face-to-face behavioral networks. *J. Theor. Biol.* **271**, 166–180 (2011).

[7] Fournet, J. & Barrat, A. Contact patterns among high school students. *PLOS ONE* **9**, e107878 (2014).

[8] Génois, M., Vestergaard, C. L., Fournet, J., Panisson, A., Bonmarin, I. & Barrat, A. Data on face-to-face contacts in an office building suggest a low-cost vaccination strategy based on community linkers. *Netw. Sci.* **3**, 326–347 (2015).

[9] Vanhems, P., Barrat, A., Cattuto, C., Pinton, J.-F., Khanafer, N., Régis, C., Kim, B.-a., Comte, B. & Voirin, N. Estimating potential infection transmission routes in hospital wards using wearable proximity sensors. *PLOS ONE* **8**, e73970 (2013).
