# Supplementary material for: Temporality modulates the effect of network heterogeneity on cooperation fixation
Source: Nat Commun. 2026 May 8;17:6238. doi: 10.1038/s41467-026-72717-w (PMC13369883; doi:10.1038/s41467-026-72717-w)
Supplement: Supplementary file 3 — Transparent Peer Review file [file 41467_2026_72717_MOESM3_ESM.pdf]

nature portfolio

Peer Review File

Temporality modulates the effect of network heterogeneity on cooperation fixation

Corresponding Author: Professor Aming Li

This file contains all reviewer reports in order by version, followed by all author rebuttals in order by version.

Version 0:

Reviewer comments:

Reviewer #1

(Remarks to the Author)
Summary

We are grateful for this opportunity to review an interesting and insightful manuscript. While appreciating the potential impact and value of the framework developed by the authors, we find that there are several important points that need further clarification/justification.
Marco Colnaghi
Gabriella Dantas Franco

1. What are the noteworthy results?
The authors establish a new framework to study the evolution of cooperative strategies in temporal networks, where only a fraction of connections between nodes is active at a given time. The most noteworthy result is that the effect of heterogeneity on the evolutionary dynamics of cooperation is determined by the rate of strategy update relative to the rate of network update. When strategy update is slow (e.g., 1000 updates per network change), this makes heterogeneity beneficial. When strategy update is fast (e.g., 10000 updates per network change), less heterogeneous networks (e.g., random regular graphs) facilitate the diffusion of cooperation better than heterogeneous ones. The latter scenario is very similar to well-known results obtained for static networks, which are recovered as a limit case.

2. Will the work be of significance to the field and related fields? How does it compare to the established literature? If the work is not original, please provide relevant references.

The role of heterogeneity in shaping the dynamics of cooperation is debated in the literature. As the authors point out, in static networks, heterogeneity enhances the equilibrium frequency of cooperation while decreasing its fixation probability. The present manuscript identifies a range of temporal dynamics under which heterogeneity actually increases cooperation's fixation probability. This result is novel and will enrich the field's understanding of how network structure shapes the evolutionary dynamics of cooperation.
To benefit cooperation researchers from other fields and the broader readership of Nature Communications, that the manuscript should include more introductory information on temporal networks and a more in-depth discussion of this model usefulness to understand real-world systems.

3. Does the work support the conclusions and claims, or is additional evidence needed?
The work presented here supports the authors' claims and conclusions. No further evidence is needed.

4. Are there any flaws in the data analysis, interpretation and conclusions? Do these prohibit publication or require revision?
The authors provide convincing arguments to support their conclusions. The mathematical framework they develop, and the results presented here, strongly support the manuscript's claims.
However, we are left with the question of whether temporal networks are the optimal model to study cooperation in the real world. While the authors do introduce the relevance of temporal networks, this introduction could be more comprehensive and in-depth. The authors make a relevant claim that natural systems may display competition and reproduction at a faster time scale than the one in which community structure changes, but the references provided for this are theoretical and do not seem to strongly support this claim. Additionally, many systems present an opposite relation between time scales (e.g.

online social networks, in which structural changes tend to be faster than strategy/opinion update). Given the interdisciplinary nature of Nature Communication, the manuscript would benefit from a more in-depth discussion of the relevance of temporal networks as a realistic model of empirical networks (see comment below) and a more in-depth discussion of non-static networks.

5. Is the methodology sound? Does the work meet the expected standards in your field?
Overall, the methodology is sound and meets the expected standards in this field. However, certain aspects of the model need further clarification (see comment below).

6. Is there enough detail provided in the methods for the work to be reproduced?
Yes – with the exception of a few points that need further clarification.

Major considerations

- The model presented here relies on the assumption that each subnetwork is a random sample of the underlying network. Can the empirical dataset be used to investigate how robust this assumption is? We would expect that, in real-world networks, a small fractions of edge change, while most of the network remains the same (for example, at a conference, a person will likely interact repeatedly with colleagues from their department – stable edges – and once or twice with many different people from other institutions – rewiring).

- With this in mind, it seems crucial to understand how temporal networks differ from dynamically changing networks, where existing links between nodes can be broken and new links created. How would a dynamical process where only a fraction of existing nodes breaks down every g steps and new connections are made compared with the “snapshot” dynamics analyzed in the paper? We would be very keen to find out whether the results generalize to a broader range of network dynamics. The model also looks very similar to ‘activity-driven’ models, in which an underlying fully connected structure is assumed, and dynamics is sampled given a distribution of ‘activity potentials’ of nodes. It would be very relevant to the field to add a comment on this as well.

- Do the authors use edge weights w_ij of the full network to calculate the expected change in cooperation frequency in the subnetwork’s largest component? Or are the w_ij’s the weights from the largest component? This is potentially confusing and should be clarified.

- It is unclear how Equation (5) has been derived (is it just an ansatz based on the simulation outcomes?) and how accurate the estimates of the transition coefficients are. Looking at the three central rightmost panels of supplementary Fig 10, it would seem that the discrepancy between estimate and simulations is higher when g is large. However, looking at the left and central panels, this does not seem to be the case. For example, looking at panels c: the theoretical transition probability from N/2 to 0 and N is around 0.5 in the left and central graphs, while it plummets to 0 in the rightmost one. Why this discrepancy?

- The authors estimate the Markov transition coefficients T_m^g (l,h) under the approximation that they are fully determined by the evolutionary dynamics in the main component of the subnetwork. How well does this assumption hold when the subnetwork is sparse? Supplementary figure 3 shows that the first largest component indeed comprises most of the population for the analyzed range of p, but for smaller values the main component might be less sizable, potentially leading to a poorer agreement between theory and simulations.
Additionally, the authors also assume that cooperators are randomly distributed (which might not be the case, given that changes in strategies likely depend on a node’s properties). To ensure the robustness of formula for the fixation probability of cooperation, it could be helpful to include a supplementary figure showing how the discrepancy between theory and simulation varies with p.

Before reviewing this paper, we were unfamiliar with the concept of temporal networks, where subnetworks are sampled from an unchanging underlying network (previously, we had only seen models where existing edges are rewired). It took us a while to understand how the rewiring of the network was taking place – indeed, that it was not really a rewiring but rather a re-sampling of an underlying network. It would help unfamiliar readers to define this term both in the abstract and the introduction; also, Figure 1 would benefit from an added panel showing the unchanging underlying network structure from which the subnetworks are sampled.

- The manuscript and the supplementary material would benefit from editing and proofreading (there are many typos and sentences that could be rewritten in a clearer form; some examples are given below).

Minor remarks

- The authors only consider the donation game, a very specific form of Prisoner’s Dilemma; how would the results generalize to a broader space of games?

- Figure legends should include all parameter values used.

- How does fixation probability change with g? From Fig. 2, one would expect that lower g would benefit cooperation, given a certain b⁄c ratio. However, in Supplementary Fig. 9 that, when g=1 and p=0.3, a very high b⁄c is needed for cooperation to be advantageous. Why is that?

Additional comments/corrections:
Manuscript:
- l. 48: Experimental studies do exist; see, for example, Gracia-Lázaro et al., PNAS 2012, and references therein.
- l. 276: N should be N_m
- l. 283: There is no need to define x_i again. π_i, however, has not yet been defined.
- eq. (3): If the edge density used is from the largest connected component, all N should be N_m.
- Similarly, all N in section "Trade-off between fixation probability and fixation time" should be N_m
- l. 280: w_ij tilde could be defined here, insted of pointing out to the supplementary material
- Last sentence of Fig. 1 is hard to interpret.
- On Fig. 3 the time axis seems redundant, as there is a title for each network presenting the snapshot number. I understand this was done to differentiate panels (a) from (b), but there's a lot of information in this figure and it would benefit from some restructuring.
- l. 129: typo 'spare' should be 'sparse'
- l. 278: typo 'oen' should be 'own'
- l. 160: Last sentences would benefit from rewriting for clarity.

Supplementary Information:
- SI Formula (1). It seems to me that this way of updating node states might benefit cooperation more than other update rules, such as pairwise comparison and birth-death process. The reasoning is as follows. Consider a cooperator surrounded by defectors – its fitness will be lower than its neighbors', so it will easily switch to defection. By contrast, if there is a defector surrounded by cooperators, its fitness will be significantly higher. With a pairwise selection process, it is unlikely that it will imitate any of its neighbors. However, using function SI (1), it seems to me that the probability that i imitates one of its neighbors increases with the number of neighbors and edges weights. Could you kindly clarify whether my intuition is correct? If it is so, it would mean that individuals with more social connections would be more susceptible to imitating their neighbors, an assumption that – while not unreasonable – should be justified more clearly. How would your results qualitatively differ if you employed a different update method, say pairwise comparison or birth-death process?
- In the paragraph after equation (2): using equation (1), should there not be a 1/N factor in the formula for e_ij^0? The 1/N term seems to be missing (the following equation is correct nevertheless, as the two 1/N terms cancel each other out).
- Equation 5: according to our calculations, the p_ij^(2) term should be p_ij^(2) tilde.
- At the beginning of page 5: when expressing P_ij^CWR in terms of P_ij (τ), the authors perform a rescaling of time units from t to 2 t/N. The reason behind this choice is unclear and could be better motivated.
- Equation 6: the p_ij coefficient in the last sum has become p_ij tilde. Please double check whether this is correct (according to our calculations, it should be p_ij). Also in equation 9, is there a missing superscript?

Reviewer #2

(Remarks to the Author)
This manuscript reports a very interesting phenomenon on evolution of cooperation in temporal structured populations. It suggests that appropriate set of heterogenous temporal structures can promote the emergence of collective cooperation, which is contrary to the conventional belief that heterogenous static structures somewhat hinder the collective cooperation. The manuscript not only shows numerically the conditions for emergence of this interesting phenomenon, but also uses quite a few empirical examples to support its findings. I believe that the results presented in this work could further complement the knowledge that distinguish the roles of the temporal and the static structures in cooperation dynamics and other networked dynamics. I therefore suggest the Editors to accept this work after the authors address the following concerns.

1. The generality of the current work: it seems that some analytical estimations and approximations have been done for the static network; however, it is not clear that the current results are valid rigorously for the temporal networks settings. More illustrative arguments and approximating results for specific networks are suggested to provide.

2. The mixture of the different types of subnetworks: the current work mainly uses the uniform types of networks as the candidates of the temporal structures. Is it possible to consider some kind of mixture of different types of subnetworks, e.g. a period of scale-free networks and then a period of regular random networks, and then their alternate appearance is taken into account? Different period proportions probably play different roles in evolution of cooperation dynamics.

3. In study of the traditional switching systems, people usually use the average system as the basis system to derive some stability or instability criteria. Actually, in the current work, the temporal networks used here are of standard switching configurations. Is it possible to derive analogous criteria where the average cooperation dynamics are used?

4. From the current numerical results, it could be seems that the larger the timescale g, the smaller the benefit-to-cost. Are their relation monotonic or non-monotonic? More justifications are welcome.

Reviewer #3

(Remarks to the Author)

I co-reviewed this manuscript with one of the reviewers who provided the listed reports. This is part of the Nature Communications initiative to facilitate training in peer review and to provide appropriate recognition for Early Career Researchers who co-review manuscripts.

Version 1:

Reviewer comments:

Reviewer #2

(Remarks to the Author)
The revision has completely addressed my major concerns raised in the first round of review. Also I notice that the authors also addressed all the comments and even critiques from the other referee. I feel that the revised manuscript is satisfactory, and could be accepted by NC. Congratulations.

(Remarks on code availability)

Reviewer #4

(Remarks to the Author)
Thank you for providing me the opportunity to read and comment upon the revised version of NCOMMS-25-33686A, “Temporality modulates the effect of network heterogeneity on cooperation fixation”. As a reviewer recruited after the first round of comments and revisions, I focus my report on both the manuscript overall and the revisions. The overall body of work, as I explain below, warrants publication and my only suggestion is that the authors address some minor writing matters related to the manuscript.

The manuscript reports a new approach to studying how cooperation evolves in temporal networks. The approach samples edges in a baseline static network to form a sequence of subnetworks and studies strategy spread in these subnetworks. In so doing, the approach yields a compelling portrayal of fleeting social encounters and that attractive substantive representation also has the virtue of preserving the baseline population structure. Using this paradigm, the authors uncover conditions in which heterogeneous temporal network structure can increase the fixation probability of cooperation, thus defying past results suggesting that network heterogeneity lessens the fixation probability of cooperators. The authors then show that empirical temporal networks would facilitate cooperation’s evolution in such a manner, and they illuminate the underlying mechanisms by explaining a tradeoff between fixation probability and fixation time as well as the influence of time scale.

The revisions add to the richness of these findings. Among a number of sound comments, Reviewer #1 noted that the authors’ novel framework substitutes for rewiring and activity-driven models, respectively, which also provide a basis for understanding how network phenomena change when only a subset of edges are made to persist. In response to this comment, the authors repeated their investigation in, respectively, a rewiring model and an activity-driven model, finding that their key conclusion concerning the effect of heterogeneity remained robust across the two alternative modeling paradigms. In a similar vein, Reviewer #1 noted that the original version of the manuscript solely focused on the donor-recipient variant of the prisoner’s dilemma, thus limiting the generality of the findings; in response, the authors have extended the work meaningfully to consider the snow-drift game. Alongside these substantial changes that added to the robustness of their findings, the authors also implemented a range of suggestions from Reviewers #1 and #2, which varied from correcting notational mishaps to elaborating on points in the discussion section. Instead of enumerating all of those changes here, I simply would attest that the authors judiciously responded to all of the reviewers’ comments listed in their response letter.

The only suggestion that I believe continues to warrant some attention concerns the writing in the manuscript. Reviewer #1 raised this concern and I believe it still holds. The paper contains typos (e.g., the authors use the word “consisting” on page 7 when intending to use the word “consistent”) and the writing at times defies grammatical conventions in ways that might impede reader comprehension of the valuable science that the manuscript reports. For instance, the second sentence of the abstract states “While previous findings suggest that structural heterogeneity in static networks hinders cooperation, real-world interactions in most natural and social systems are dynamic and best represented as temporal networks”; please note that this excerpt is an example of “faulty subordination” in the sense that the subordinate clause (“While … cooperation,”) does not align with the main clause which focuses on real-world interaction whereas the subordinate clause focuses on findings. Such issues occur throughout the paper and might impede reader comprehension. Addressing these minor matters would ensure that the quality science at the heart of the paper is not clouded by correctable writing issues.

But, again, those are very minor matters that should not prevent publication. The paper presents a new modeling framework that will prove to be influential in the study of temporal networks and social evolution, and it uses this framework in an investigation that yields an important finding that overturns the field's understanding of how network heterogeneity affects cooperation.

(Remarks on code availability)

Open Access This Peer Review File is licensed under a Creative Commons Attribution 4.0 International License, which permits use, sharing, adaptation, distribution and reproduction in any medium or format, as long as you give appropriate credit to the original author(s) and the source, provide a link to the Creative Commons license, and indicate if changes were made.
In cases where reviewers are anonymous, credit should be given to 'Anonymous Referee' and the source.
The images or other third party material in this Peer Review File are included in the article's Creative Commons license, unless indicated otherwise in a credit line to the material. If material is not included in the article's Creative Commons license and your intended use is not permitted by statutory regulation or exceeds the permitted use, you will need to obtain permission directly from the copyright holder.
To view a copy of this license, visit https://creativecommons.org/licenses/by/4.0/

Response to Reviewer #1:

Summary

We are grateful for this opportunity to review an interesting and insightful manuscript. While appreciating the potential impact and value of the framework developed by the authors, we find that there are several important points that need further clarification/justification.
Marco Colnaghi
Gabriella Dantas Franco

We thank you for reviewing our manuscript and pointing out the significance of our study to related fields. We next address each of the comments in order.

1. What are the noteworthy results?
The authors establish a new framework to study the evolution of cooperative strategies in temporal networks, where only a fraction of connections between nodes is active at a given time. The most noteworthy result is that the effect of heterogeneity on the evolutionary dynamics of cooperation is determined by the rate of strategy update relative to the rate of network update. When strategy update is slow (e.g., 1000 updates per network change), this makes heterogeneity beneficial. When strategy update is fast (e.g., 10000 updates per network change), less heterogeneous networks (e.g., random regular graphs) facilitate the diffusion of cooperation better than heterogeneous ones. The latter scenario is very similar to well-known results obtained for static networks, which are recovered as a limit case.

We thank you for the positive assessment of our main results.

2. Will the work be of significance to the field and related fields? How does it compare to the established literature? If the work is not original, please provide relevant references.

The role of heterogeneity in shaping the dynamics of cooperation is debated in the literature. As the authors point out, in static networks, heterogeneity enhances the equilibrium frequency of cooperation while decreasing its fixation probability. The present manuscript identifies a range of temporal dynamics under which heterogeneity actually increases cooperation's fixation probability. This result is novel and will enrich the field's understanding of how network structure shapes the evolutionary dynamics of cooperation.
To benefit cooperation researchers from other fields and the broader readership of Nature Communications, that the manuscript should include more introductory information on temporal networks and a more in-depth discussion of this model usefulness to understand real-world systems.

We thank you for highlighting the novelty and significance of our findings. We have added more background on temporal networks and expanded the introduction of their relevance for real-world systems by adding *"A key feature of such temporal networks is that interactions are not continuously present but instead appear and disappear over time, leading to intermittent links that static structures cannot capture [27, 29]. Such temporality has been shown to strongly influence dynamical processes such as epidemic spreading, information diffusion, and synchronization [29, 30]. In evolutionary game theory, temporal structures introduce a novel dimension of*

heterogeneity: not only who interacts with whom matters, but also when interactions occur. Recent studies suggest that the duration of temporal interactions can fundamentally reshape cooperative dynamics beyond what is observed on time-aggregated static networks [15, 31]. Therefore, understanding the role of temporal networks in the evolution of cooperation is crucial for bridging theoretical predictions with realistic, time-varying social and biological systems." to lines 52-62 in the Introduction of the revised manuscript.

3. Does the work support the conclusions and claims, or is additional evidence needed?
The work presented here supports the authors' claims and conclusions. No further evidence is needed.

We thank you for confirming that our results fully support the claims and conclusions.

4. Are there any flaws in the data analysis, interpretation and conclusions? Do these prohibit publication or require revision?
The authors provide convincing arguments to support their conclusions. The mathematical framework they develop, and the results presented here, strongly support the manuscript's claims. However, we are left with the question of whether temporal networks are the optimal model to study cooperation in the real world. While the authors do introduce the relevance of temporal networks, this introduction could be more comprehensive and in-depth. The authors make a relevant claim that natural systems may display competition and reproduction at a faster time scale than the one in which community structure changes, but the references provided for this are theoretical and do not seem to strongly support this claim. Additionally, many systems present an opposite relation between time scales (e.g. online social networks, in which structural changes tend to be faster than strategy/opinion update). Given the interdisciplinary nature of Nature Communication, the manuscript would benefit from a more in-depth discussion of the relevance of temporal networks as a realistic model of empirical networks (see comment below) and a more in-depth discussion of non-static networks.

We thank you for recognizing the strength of our analysis and conclusions. We have replaced the references with empirical studies ([33, 34] in the revised manuscript). We have also added a new paragraph in Discussion section (see the detailed response to the first comment in "Major considerations") to provide a more in-depth discussion of synthetic models in understanding realistic temporal networks.

5. Is the methodology sound? Does the work meet the expected standards in your field?
Overall, the methodology is sound and meets the expected standards in this field. However, certain aspects of the model need further clarification (see comment below).

We thank you for confirming that our methodology is sound and meets the standards of the field. We have clarified the specific aspects of the model as noted in the detailed responses below.

6. Is there enough detail provided in the methods for the work to be reproduced?
Yes – with the exception of a few points that need further clarification.

We thank you for confirming that the methods are generally sufficient for reproducibility. We have clarified the few points noted, as detailed in our responses to specific comments below.

Major considerations

- The model presented here relies on the assumption that each subnetwork is a random sample of the underlying network. Can the empirical dataset be used to investigate how robust this assumption is? We would expect that, in real-world networks, a small fractions of edge change, while most of the network remains the same (for example, at a conference, a person will likely interact repeatedly with colleagues from their department – stable edges – and once or twice with many different people from other institutions – rewiring).

- With this in mind, it seems crucial to understand how temporal networks differ from dynamically changing networks, where existing links between nodes can be broken and new links created. How would a dynamical process where only a fraction of existing nodes breaks down every g steps and new connections are made compared with the "snapshot" dynamics analyzed in the paper? We would be very keen to find out whether the results generalize to a broader range of network dynamics. The model also looks very similar to 'activity-driven' models, in which an underlying fully connected structure is assumed, and dynamics is sampled given a distribution of 'activity potentials' of nodes. It would be very relevant to the field to add a comment on this as well.

We thank you for your insightful comments and proposing this interesting modelling framework. We constructed a rewiring model starting from a synthetic scale-free network, where a fraction p_N of nodes is selected and their links are rewired to other nodes with probability p_E every g steps.

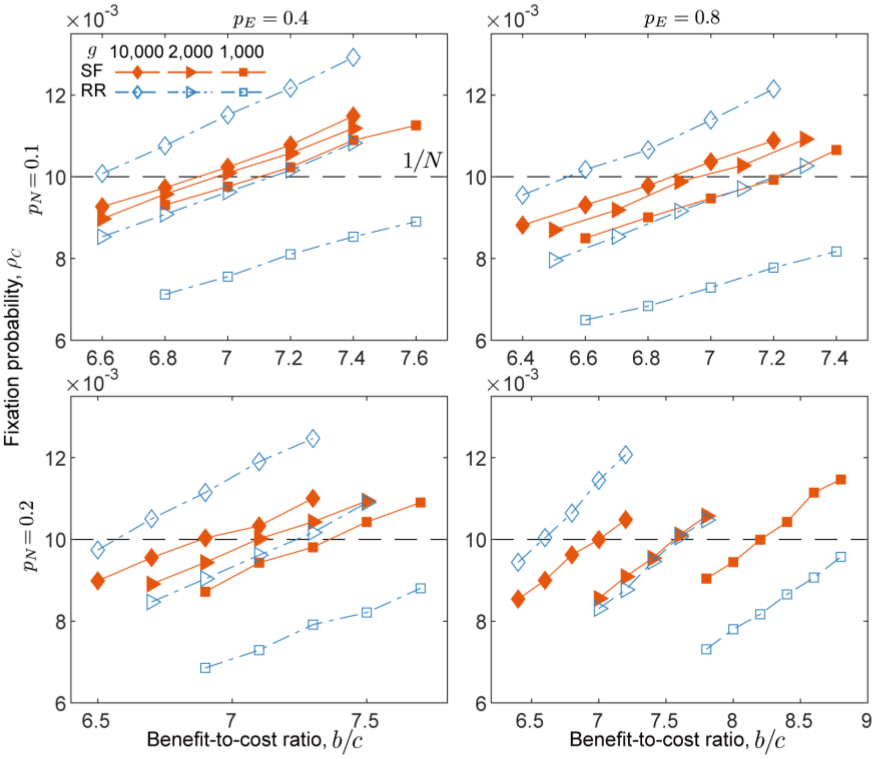

Figure R1: Fixation of cooperation on dynamic networks constructed by the rewiring model. We generate the dynamic networks using the rewiring model starting from a scale-free network. We numerically

calculate the fixation probability of cooperation across different network parameters ($p_N = 0.1, 0.2$; $p_E = 0.4, 0.8$) and evolutionary timescale $g = 1000, 2000, 10000$. As a comparison, we numerically calculate the fixation probability on the corresponding random regular networks as control groups. We show that, for all network parameter settings, when the evolutionary timescale g is relatively small, the heterogeneous dynamic networks promote cooperation than homogeneous counterparts, despite that only a small proportion of nodes break and rewire. However, temporal random regular networks show a higher fixation probability when $g = 10000$. This result implies that, with sufficient time of evolution, cooperative clusters can be formed and sustained even on random regular networks, which reduces the advantage of heterogeneous networks caused by shorter fixation times.

Moreover, we have calculated the fixation probability on temporal networks generated by the rewiring model across different evolutionary timescales, and compared with those for homogeneous counterparts (Fig. R1). Our findings confirm the main conclusion: heterogeneous networks maintain an advantage through shorter fixation times, but this advantage vanishes as g increases. These results demonstrate that our conclusions generalize to a broader class of network modelling approaches.

Besides, we appreciate your insightful comment on the activity-driven model, which indeed share the similarity with our proposed temporal networks. To show the robustness of our results, we implemented simulations on the activity-driven model with a power-law activity distribution, and compared results with their homogeneous counterparts. We obtained the consistent results with that on our proposed model. These results also show that the advantage of heterogeneous temporal networks shrinks as the networks become denser.

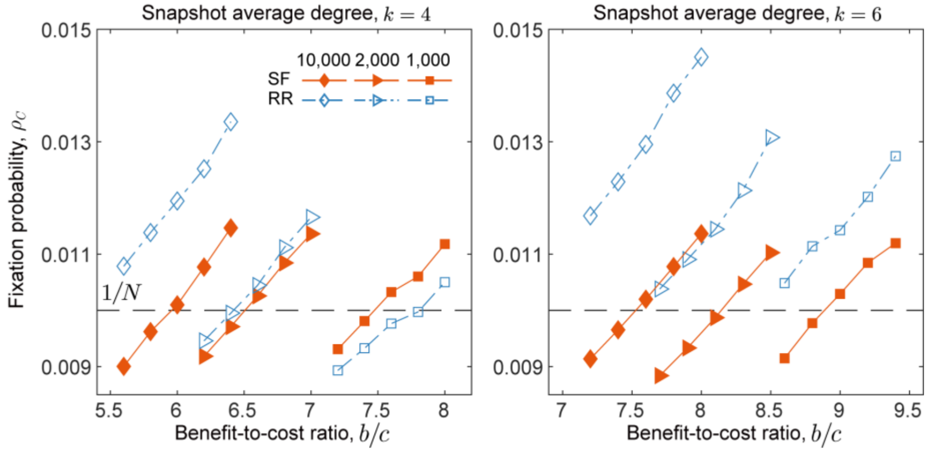

Figure R2: Fixation of cooperation on temporal networks constructed by the activity-driven model. We generate the temporal networks using the activity-driven model with a power-law activity distribution. We numerically calculate the fixation probability of cooperation across different snapshot average degree ($k = 4, 6$) and evolutionary timescale $g = 1000, 2000, 10000$. As a comparison, we numerically calculate the fixation probability on the corresponding random regular networks as control groups. We show that, for sparse networks ($k = 4$), when the evolutionary timescale g is relatively small, the heterogeneous dynamic networks promote cooperation than homogeneous counterparts. In contrast, for dense networks ($k = 6$), the homogeneous networks outperform the heterogeneous temporal networks in promoting the fixation of cooperation across the three values of g, which is consistent with our results on both synthetic models and empirical networks (Fig. 3 in the main text).

Motivated by your constructive comments, we have added two paragraphs (as below) to show the robustness of our results across different modelling approach in the end of the first subsection in Results. We have also added Figs. R1 and R2 to Supplementary Figs. S7–S8.

"To assess the generality of our results for a broader class of dynamic network models, we implemented a rewiring model in which, after every g evolutionary steps, a fraction p_N of nodes is selected, and each of their links is rewired with probability p_E to another randomly chosen node. We calculated the fixation probability of cooperation across a range of parameter settings for this rewiring model starting from a scale-free network, and compared the results with their homogeneous counterparts (Supplementary Fig. S7). We confirm our main conclusion—heterogeneous networks maintain an advantage through shorter fixation times, but this advantage gradually diminishes as the timescale g increases.

In addition, we considered an activity-driven model in which nodes are assigned activity potentials drawn from a power-law distribution, and at each time step, active nodes form temporary connections with randomly chosen peers [36]. Simulations on this model show results consistent with those obtained from both our proposed random subnetwork model and the node-rewiring model (Supplementary Fig. S8). In particular, for sparse networks, the advantage of heterogeneous temporal networks in facilitating cooperation diminishes as g increases. Yet for dense networks, the homogeneous networks outperform the heterogeneous temporal networks in promoting the fixation of cooperation across all values of g examined. These results further support the robustness and generality of our findings across different temporal network frameworks."

In addition, we have added a new paragraph (as below) in Discussion to provide a more in-depth discussion of synthetic models in understanding realistic temporal networks.

"Different network models capture different aspects of reality. The random sampling models can describe situations where interactions are randomly drawn in each period. The activity-driven model also draws interactions randomly on top of an effective substrate network but emphasizes random node activations. Rewiring models are more appropriate for systems in which most interactions persist but occasionally change. While these models highlight different empirical scenarios, our main conclusions remain valid across synthetic models and empirical datasets (Figs. 2 and 3, Supplementary Figs. 7 and 8). Future work may further bridge synthetic frameworks and empirical systems through hybrid or data-driven temporal models."

- Do the authors use edge weights w_ij of the full network to calculate the expected change in cooperation frequency in the subnetwork's largest component? Or are the w_ij's the weights from the largest component? This is potentially confusing and should be clarified.

Thanks for pointing out this potential confusion. We used the edge weights w_{ij} to indicate the weights from the largest component. Following this suggestion, we have clarified this point in Methods.

- It is unclear how Equation (5) has been derived (is it just an ansatz based on the simulation outcomes?) and how accurate the estimates of the transition coefficients are. Looking at the three central rightmost panels of supplementary Fig 10, it would seem that the discrepancy between

estimate and simulations is higher when g is large. However, looking at the left and central panels, this does not seem to be the case. For example, looking at panels c: the theoretical transition probability from N/2 to 0 and N is around 0.5 in the left and central graphs, while it plummets to 0 in the rightmost one. Why this discrepancy?

Thanks again for careful observations. Equation (5) is obtained directly on the simulation outcomes to capture the dominant tendency in the probability distribution during the evolution. To clarify the potential confusion in Supplementary Fig. 16c (in the revised version): in the left and central panels, the theoretical transition probability from $N/2$ to 0 and N approaches 0.5 as g tends to infinity (e.g., $g = 10^5$). In contrast, the right panel only shows cases of $g = 10^3, 2 \times 10^3$, and 10^4, which correspond to the leftmost values on the x-axis in the left and central panels. Therefore, the probability is expected to remain small and close to 0.

- The authors estimate the Markov transition coefficients T_m^g (l,h) under the approximation that they are fully determined by the evolutionary dynamics in the main component of the subnetwork. How well does this assumption hold when the subnetwork is sparse? Supplementary figure 3 shows that the first largest component indeed comprises most of the population for the analyzed range of p, but for smaller values the main component might be less sizable, potentially leading to a poorer agreement between theory and simulations.
Additionally, the authors also assume that cooperators are randomly distributed (which might not be the case, given that changes in strategies likely depend on a node's properties). To ensure the robustness of formula for the fixation probability of cooperation, it could be helpful to include a supplementary figure showing how the discrepancy between theory and simulation varies with p.

Thanks for this constructive suggestion. We fully agree that both the sparsity of the networks and the local distribution of cooperators may affect the simulation results. To show the robustness of our assumptions and how the discrepancy between theory and simulation varies with p, we have calculated the theoretical values of critical benefit-to-cost ratio $(b/c)^*$ (Fig. R3), and implemented corresponding numerical simulations of synthetic heterogenous temporal networks and their homogeneous counterparts (Fig. R4).

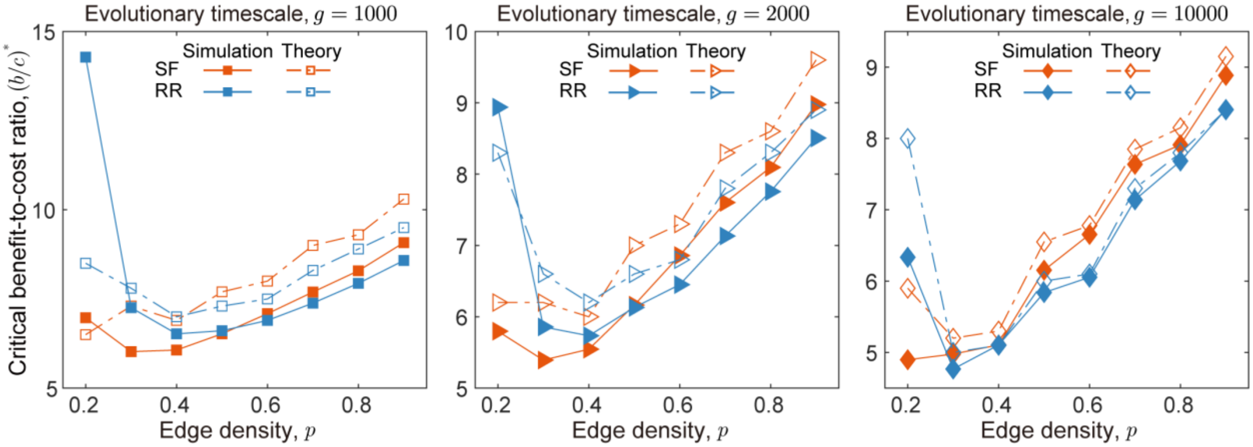

Figure R3: Comparison of critical benefit-to-cost ratio between numerical and theoretical results. We theoretically calculate the critical benefit-to-cost ratio on synthetic temporal scale-free and random regular networks with edges density p from 0.2 to 0.9, and compare it with the numerical results. We show that our theoretical calculations remain valid across different evolutionary timescales ($g = 1000, 2000, 10000$),

although the discrepancy increases when the networks are relatively sparse. In the case of large evolutionary timescale g (e.g., $g = 10000$), our theoretical predictions present remarkable accuracy as edge density p increases.

We show that our theoretical calculations remain valid across different evolutionary timescales in Fig. R3, even when the edge density p is low. However, when the subnetwork is extremely sparse (e.g., $p = 0.1$), the synthetic temporal scale-free networks require a significantly large benefit-to-cost ratio, which making the weak selection condition underlying the theoretical derivations no longer applicable (Fig. R4). On the other hand, the synthetic random regular networks are difficult to promote the fixation of cooperation ($\rho_C < 1/N$ across all evolutionary timescale g), since achieving connectivity is difficult even in the aggregated network, which in turn inhibits the spread of cooperation.

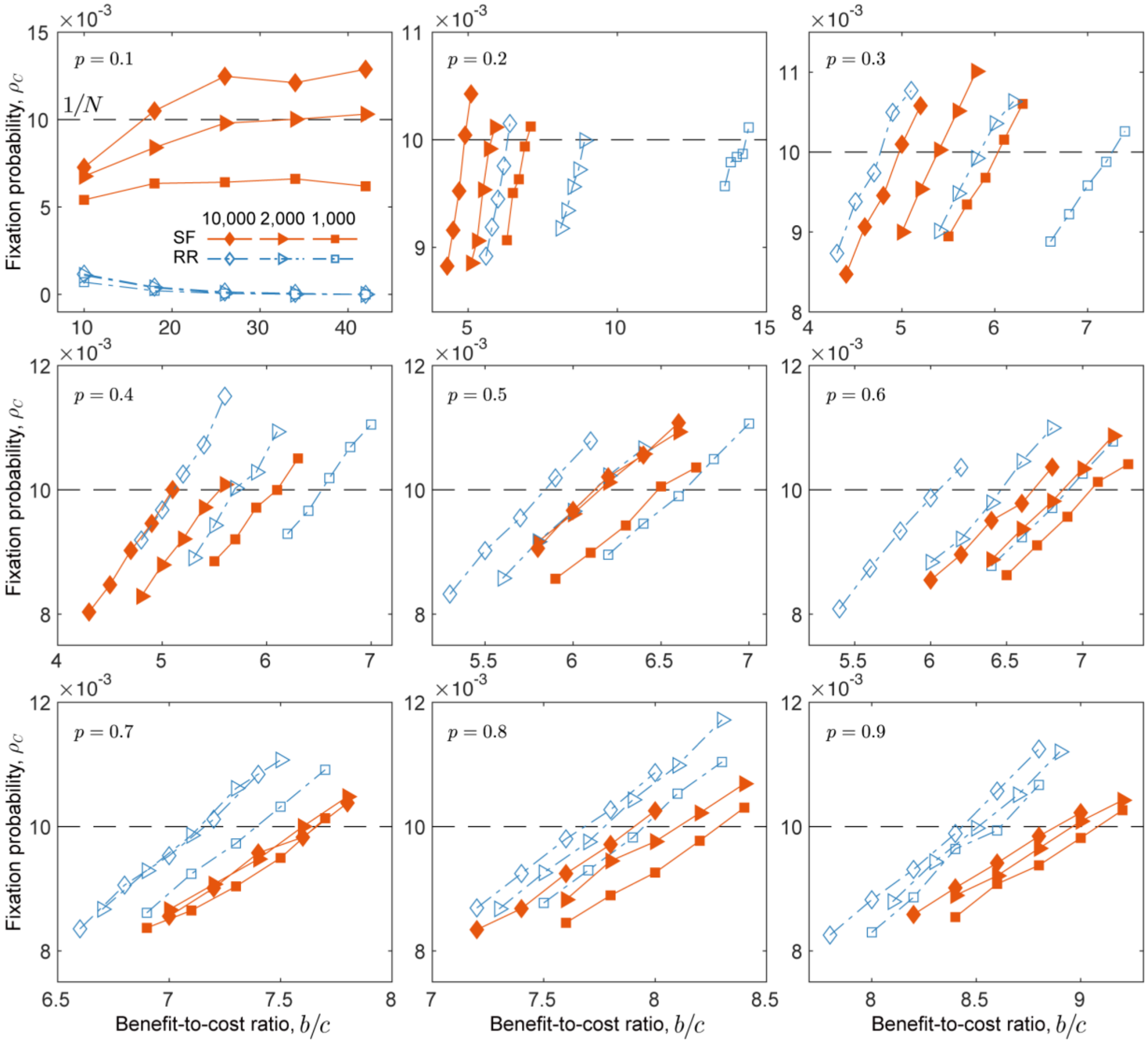

Figure R4: Fixation of cooperation on synthetic temporal scale-free and random regular networks. We generate the synthetic temporal networks with edge density p from 0.1 to 0.9, and numerically calculate the fixation probability of cooperation across different evolutionary timescale $g = 1000, 2000, 10000$. The network size is $N = 100$. The other parameters are the same as those used in Fig. 2 in the main text.

Motivated by this constructive comment, we have added Figs. R3 and R4 to Supplementary Figs. S4 and S5 in Supplementary Information, and added *"We show that our theoretical results remain valid across a wide range of edge density p, even in the case that the subnetworks are relatively sparse (Supplementary Fig. S4)."* to lines 395-396 in the revised main text.

Before reviewing this paper, we were unfamiliar with the concept of temporal networks, where subnetworks are sampled from an unchanging underlying network (previously, we had only seen models where existing edges are rewired). It took us a while to understand how the rewiring of the network was taking place – indeed, that it was not really a rewiring but rather a re-sampling of an underlying network. It would help unfamiliar readers to define this term both in the abstract and the introduction; also, Figure 1 would benefit from an added panel showing the unchanging underlying network structure from which the subnetworks are sampled.

We thank you for this very constructive comment. We fully agree that additional explanation and definition should be provided to appeal to a broader readership. Cosidering that our results demonstrate robustness on synthetic temporal networks generated from different models (e.g., re-sampling, rewiring, and the activity-driven model), as well as the real dataset, we feel it is more appropriate to clarify the modelling framework at the beginning of the Results Section by adding an paragraph (as below), in order to avoid any misunderstanding that our conclusions are tied to a specific setting.

"Here we consider temporal networks as sequences of subnetworks sampled from an underlying static network. At every g generations of evolutionary dynamics, a subnetwork is generated by randomly selecting a fraction of edges from the underlying network, while the set of nodes remains unchanged (Fig. 2a). This random resampling framework allows us to model fluctuating interactions without altering the overall population structure. Importantly, we also consider alternative generative models, including node-rewiring dynamics and activity-driven networks, to ensure that our findings are robust across different temporal network constructions. By comparing the fixation probabilities of cooperation under these different settings, we have confirmed that the main conclusions are not specific to any single modelling choice."

In line with this consideration, we have also adopted your constructive suggestion and added a schematic panel in Fig. 2a (please see the revised main text) to illustrate how subnetworks are sampled from the underlying network. At the same time, we decided to retain the original Figure 1, as it emphasizes the more general illustration of our framework—subnetworks switch after finite evolutionary time. We believe that these changes will make the concept much clearer to readers who may be less familiar with this modelling approach.

- The manuscript and the supplementary material would benefit from editing and proofreading (there are many typos and sentences that could be rewritten in a clearer form; some examples are given below).

We thank the you for your great efforts in pointing this out and have corrected all the issues accordingly.

Minor remarks

- The authors only consider the donation game, a very specific form of Prisoner's Dilemma; how would the results generalize to a broader space of games?

We thank you for this insightful point. We have extended our analysis to snowdrift game ($T = \beta, R = \beta - 1/2, S = \beta - 1, P = 0$), and we find that, when the edge density is relatively low (e.g., $p = 0.3$), temporal scale-free networks achieve a higher fixation probability (Fig. R5). When networks become denser (e.g., $p = 0.7$), the homogeneous counterpart outperform the temporal scale-free in promoting cooperation, which is consistent with our results obtained in the canonical Prisoner's Dilemma game. To show the generality of our main conclusion across different game types, we have added Fig. R5 to Supplementary Fig. S9, and added a paragraph (as below) to the end of the corresponding subsection.

"Moreover, we show that this general conclusion can be applied to different game types. We calculate the fixation probability on synthetic temporal scale-free networks compared to their homogeneous counterpart with snowdrift games ($T = \beta, R = \beta - 1/2, S = \beta - 1, P = 0$), and find that the advantage of heterogeneous networks shrinks when the networks become denser (Supplementary Fig. S9). This result indicates that the trade-off between the fixation probability and fixation time on subnetworks remains valid under different game types."

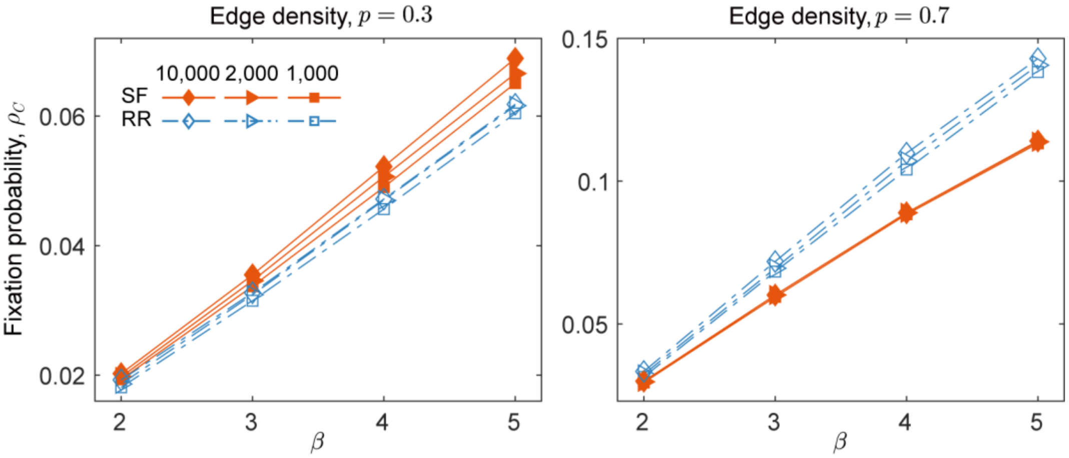

Figure R5: Fixation of cooperation on temporal networks under the snowdrift game. We numerically calculate the fixation probability of cooperation on temporal scale-free networks across different edge density ($p = 0.3, 0.7$) under a snowdrift game ($T = \beta, R = \beta - 1/2, S = \beta - 1, P = 0$). As a comparison, we numerically calculate the fixation probability on the corresponding random regular networks as control groups. We show that, when the edge density is low ($p = 0.3$), the heterogeneous temporal networks promote cooperation more than homogeneous counterparts across all evolutionary timescale g. However, the trend reverses for dense networks ($p = 0.7$). These results are consistent with our results on both synthetic models and empirical networks (Fig. 3 in the main text). Here we generate the synthetic temporal networks with $N = 100$ and the average degree of the aggregated scale-free network is $k = 6$. The other parameters are the same as those used in Fig. 2 in the main text.

- Figure legends should include all parameter values used.

Fixed.

- How does fixation probability change with g? From Fig. 2, one would expect that lower g would benefit cooperation, given a certain b/c ratio. However, in Supplementary Fig. 9 that, when g=1 and p=0.3, a very high b/c is needed for cooperation to be advantageous. Why is that?

Thank you for this careful reading. We would like to clarify a possible misunderstanding: in Fig. 2, a lower g (e.g., $g = 1000$, square markers) actually corresponds to a higher critical benefit-to-cost ratio, which means it is less favourable for cooperation. This is consistent with the observation in Supplementary Fig. 14 in the revised version.

Additional comments/corrections:
Manuscript:
- l. 48: Experimental studies do exist; see, for example, Gracia-Lázaro et al., PNAS 2012, and references therein.
- l. 276: N should be N_m
- l. 283: There is no need to define x_i again. π_i, however, has not yet been defined.
- eq. (3): If the edge density used is from the largest connected component, all N should be N_m.
- Similarly, all N in section "Trade-off between fixation probability and fixation time" should be N_m
- l. 280: w_ij tilde could be defined here, insted of pointing out to the supplementary material
- Last sentence of Fig. 1 is hard to interpret.
- On Fig. 3 the time axis seems redundant, as there is a title for each network presenting the snapshot number. I understand this was done to differentiate panels (a) from (b), but there's a lot of information in this figure and it would benefit from some restructuring.
- l. 129: typo 'spare' should be 'sparse'
- l. 278: typo 'oen' should be 'own'
- l. 160: Last sentences would benefit from rewriting for clarity.

Thank you very much for these detailed and helpful suggestions. We have carefully revised the manuscript and supplementary material accordingly (see revised text marked in blue), correcting all typos including these ones, clarifying definitions, and restructuring figures/text where suggested.

Supplementary Information:
- SI Formula (1). It seems to me that this way of updating node states might benefit cooperation more than other update rules, such as pairwise comparison and birth-death process. The reasoning is as follows. Consider a cooperator surrounded by defectors – its fitness will be lower than its neighbors', so it will easily switch to defection. By contrast, if there is a defector surrounded by cooperators, its fitness will be significantly higher. With a pairwise selection process, it is unlikely that it will imitate any of its neighbors. However, using function SI (1), it seems to me that the probability that i imitates one of its neighbors increases with the number of neighbors and edges weights. Could you kindly clarify whether my intuition is correct? If it is so, it would mean that individuals with more social connections would be more susceptible to imitating their neighbors, an assumption that – while not unreasonable – should be justified more clearly. How would your results qualitatively differ if you employed a different update method, say pairwise comparison or birth-death process?

We thank you for this very insightful comment. The intuition is indeed correct that under imitation update rule (Supplementary Note 1)—individuals with more neighbours are more susceptible to imitating the strategy from one of its many neighbours. As you pointed out, other rules such as the birth-death process are well known to be less favourable to cooperation on static networks (see, e.g., Ohtsuki *et al.*, *Nature*, 2006), and thus we would not expect our main conclusions to qualitatively change under those dynamics.

- In the paragraph after equation (2): using equation (1), should there not be a 1/N factor in the formula for e_ij^0? The 1/N term seems to be missing (the following equation is correct nevertheless, as the two 1/N terms cancel each other out).

We thank you for pointing out this mismatch. In that paragraph, the later occurrence of e_{ij}° indeed missed the factor $1/N$. We have corrected it to $e_{ij}^{\circ} = \tilde{p}_{ji}/N$.

- Equation 5: according to our calculations, the p_ij^(2) term should be p_ij^(2) tilde.

Corrected.

- At the beginning of page 5: when expressing P_ij^CWR in terms of P_ij (τ), the authors perform a rescaling of time units from t to 2t/N. The reason behind this choice is unclear and could be better motivated.

Thanks again. We have added *"At each time step during the evolutionary process, one of the two random walkers is chosen to take a step of random walk with probability* $2/N$*. We now only consider two independent random walks starting from i and j on the replacement network with meeting time* τ*, where each of the random walkers is chosen with equal probability* $1/2$ *at each time step. Therefore, the coalescing time of two random workers during the evolutionary process chosen with probability* $1/N$ *can be approximately rescaled with that chosen with probability* $1/2$ *by a factor of* $2/N$*."* to clarify this derivation detail.

- Equation 6: the p_ij coefficient in the last sum has become p_ij tilde. Please double check whether this is correct (according to our calculations, it should be p_ij). Also in equation 9, is there a missing superscript?

Thanks again. We have corrected all corresponding typos.

In summary, we wish to thank you for their thorough and constructive review of our manuscript. The comments therein have considerably improved the paper.

Response to Reviewer #2:

This manuscript reports a very interesting phenomenon on evolution of cooperation in temporal structured populations. It suggests that appropriate set of heterogenous temporal structures can promote the emergence of collective cooperation, which is contrary to the conventional belief that heterogenous static structures somewhat hinder the collective cooperation. The manuscript not only shows numerically the conditions for emergence of this interesting phenomenon, but also uses quite a few empirical examples to support its findings. I believe that the results presented in this work could further complement the knowledge that distinguish the roles of the temporal and the static structures in cooperation dynamics and other networked dynamics. I therefore suggest the Editors to accept this work after the authors address the following concerns.

We sincerely thank you for the positive and encouraging assessment of our work. We are pleased that you recognize the novelty and significance of our results, and we have carefully addressed all the concerns raised below.

1. The generality of the current work: it seems that some analytical estimations and approximations have been done for the static network; however, it is not clear that the current results are valid rigorously for the temporal networks settings. More illustrative arguments and approximating results for specific networks are suggested to provide.

We thank you for this helpful suggestion. In the revised version, to further illustrate the generality of our results, we have conducted new simulations and approximate theoretical calculations for heterogeneous temporal networks generated with edge sampling probabilities p ranging from 0.1 to 0.9, together with homogeneous network counterparts for comparison. These results are provided in Supplementary Figs. S4 and S5 (Figs. R3 and R4), demonstrating that our main conclusions hold across a broad range of temporal network settings. For our analytical estimations, we have stressed its validity in both static and temporal networks in Methods.

2. The mixture of the different types of subnetworks: the current work mainly uses the uniform types of networks as the candidates of the temporal structures. Is it possible to consider some kind of mixture of different types of subnetworks, e.g. a period of scale-free networks and then a period of regular random networks, and then their alternate appearance is taken into account? Different period proportions probably play different roles in evolution of cooperation dynamics.

We thank you for this very insightful suggestion. To address it, we constructed a new type of mixed temporal network following your suggestion, in which a fraction p_s of each evolutionary period g uses scale-free networks and the remaining fraction $1 - p_s$ uses random regular networks, with the network types alternating in successive periods. We calculated the fixation probability on the mixed temporal networks with alternative appearance of scale-free and random regular networks (grey markers in Fig. R6), compared to the fixation dynamics on temporal networks with pure appearance. We show that for different p_s values ($p_s = 0.2, 0.5, 0.8$), those mixed temporal networks present the lowest fixation probability compared to the pure appearance for all evolutionary timescale. This also lies in the form of cooperative clusters during the course of evolution—the switching between different network models undermines the structural similarity

preserved in subnetworks sampled from the same underlying network, thereby leading to a more pronounced breakdown of cooperative clusters.

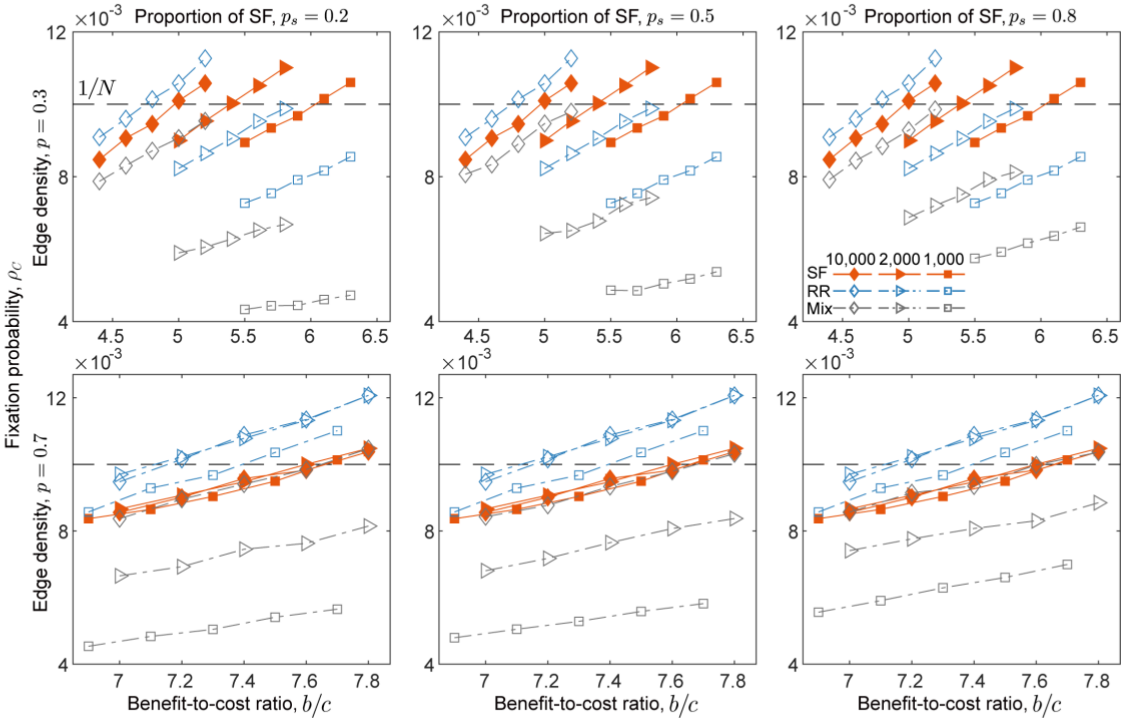

Figure R6: Fixation of cooperation on temporal networks with alternate appearance of scale-free and random regular networks. We construct a mixed temporal network in which a fraction p_s of each evolutionary period g uses scale-free (SF) networks and the remaining fraction $1 - p_s$ uses random regular (RR) networks, with the network type alternating in successive periods. We numerically calculate the fixation probability of cooperation on the mixed temporal networks (markers in grey) across different edge density ($p = 0.3$ in the first row, $p = 0.7$ in the second row) and evolutionary timescales ($g = 10^3, 2 \times 10^3, 10^4$ in columns from the left to right panel). Then, we compare the results with those on the temporal SF (markers in orange) and RR (markers in blue) networks. We show that the mixed temporal networks present the lowest fixation probability in all parameter settings, with the least ability to promote cooperation. Here we generate the synthetic temporal networks with $N = 100$ and the average degree of the aggregated scale-free network is $k = 6$. The other parameters are the same as those used in Fig. 2 in the main text.

Motivated by this constructive comment, we have added Fig. R6 to Supplementary Fig. S15 in Supplementary Information, and added a new paragraph (as below) in the end of Results to further clarify the underlying mechanisms in the evolution of cooperation on temporal networks.

"Moreover, we further consider a mixed temporal network model, where a fraction p_s of each evolutionary period is constructed from scale-free networks and the remaining fraction $1 - p_s$ from random regular networks, with the two types alternating in successive periods (Supplementary Fig. S15). Compared with the case in which the subnetworks are drawn from just one static substrate network, these mixed temporal networks consistently yield the lowest fixation probability of cooperation across different evolutionary timescales. Mechanistically, this suppression effect can be attributed to the disruption of structural similarity: while subnetworks sampled from the same underlying network preserve certain topological correlations that support

the sustaining of cooperative clusters, alternating between fundamentally different network types undermines such correlations, making the emergence of cooperation more difficult."

3. In study of the traditional switching systems, people usually use the average system as the basis system to derive some stability or instability criteria. Actually, in the current work, the temporal networks used here are of standard switching configurations. Is it possible to derive analogous criteria where the average cooperation dynamics are used?

We thank you for this excellent suggestion. Indeed, the idea of using an "average system" to derive stability or average dynamics is very relevant. As noted in McAvoy *et al.* (*Nat. Hum. Behav.*, 2020), introducing stochastic randomness in cooperative interactions—where at each time step a cooperator randomly allocates benefits to neighbours—naturally leads to averaged evolutionary outcomes over time, which is equivalent to the setting where each cooperator's payoff is distributed equally among neighbours on the aggregated static network. In other words, this temporal randomness in games ultimately converges to the average cooperation dynamics on static settings. Our model, in contrast, allows sufficiently long evolution on each snapshot and constructs a switching system, thereby highlighting the distinct and intriguing dynamics arising from temporally switching networks.

Motivated by your excellent suggestion, we add a new paragraph (as below) in Discussion to point out this insightful perspective.

"In traditional studies of switching systems, average systems are often used for deriving stability or instability criteria [35, 47], and a similar question arises in our context of cooperation on temporal networks. Previous work has shown that when cooperators randomly select a recipient of a donation at each time step, the final evolutionary outcome is equivalent to a static network where contributions are evenly distributed among all possible recipients under weak selection [48]. However, previous studies have shown that when the evolution of network structures is involved, even the evolution in small scales cannot be effectively approximated by averaging methods [49, 50]. For temporal networks—where structural changes are exogenous and often exhibit bursty rhythms—it remains an open challenge to identify effective averaging approaches. We believe that our conclusions may provide a useful foundation for developing such methods in the future."

4. From the current numerical results, it could be seems that the larger the timescale \$g\$, the smaller the benefit-to-cost. Are their relation monotonic or non-monotonic? More justifications are welcome.

We thank you for raising this important question. Our results show that the fixation probability as a function of the evolutionary timescale g is generally monotonic in the snapshot model (Supplementary Fig. S16). The reason is that a larger g allows cooperative clusters to spread more fully within each subnetwork, making them more robust to subsequent network switching, which in turn lowers the critical benefit-to-cost ratio required for cooperation. However, the outcome can also depend on the specific distribution of cooperators when switching occurs and on the structural details of the successive subnetworks, which may lead to diverse patterns. Therefore, deriving a direct analytical relationship between the critical benefit-to-cost ratio and g on arbitrary temporal networks remains an open problem for future research.

Motivated by this insightful comment, we have added *"so that the critical benefit-to-cost ratio decreases monotonically with the evolutionary timescale* g*."* to lines 265 to 266, and added *"While our simulations suggest a generally monotonic effect of* g *in snapshot-based temporal network models, whether such monotonicity holds on arbitrary temporal networks remains an open question."* to lines 268-270 in the main text.

In summary, we wish to thank you for the valuable comments and suggestions. Addressing them has considerably improved the quality of our manuscript.

Response to Reviewer #3:

I co-reviewed this manuscript with one of the reviewers who provided the listed reports. This is part of the Nature Communications initiative to facilitate training in peer review and to provide appropriate recognition for Early Career Researchers who co-review manuscripts.

We wish to thank you for the tremendous efforts in co-reviewing our manuscript. These comments have markedly improved the clarity and relevance of our work to a broad audience.

Response to Reviewer #2:

The revision has completely addressed my major concerns raised in the first round of review. Also I notice that the authors also addressed all the comments and even critiques from the other referee. I feel that the revised manuscript is satisfactory, and could be accepted by NC. Congratulations.

We thank you for reviewing our manuscript again and for the previous insightful comments and suggestions.

Response to Reviewer #4:

Thank you for providing me the opportunity to read and comment upon the revised version of NCOMMS-25-33686A, “Temporality modulates the effect of network heterogeneity on cooperation fixation”. As a reviewer recruited after the first round of comments and revisions, I focus my report on both the manuscript overall and the revisions. The overall body of work, as I explain below, warrants publication and my only suggestion is that the authors address some minor writing matters related to the manuscript.

The manuscript reports a new approach to studying how cooperation evolves in temporal networks. The approach samples edges in a baseline static network to form a sequence of subnetworks and studies strategy spread in these subnetworks. In so doing, the approach yields a compelling portrayal of fleeting social encounters and that attractive substantive representation also has the virtue of preserving the baseline population structure. Using this paradigm, the authors uncover conditions in which heterogeneous temporal network structure can increase the fixation probability of cooperation, thus defying past results suggesting that network heterogeneity lessens the fixation probability of cooperators. The authors then show that empirical temporal networks would facilitate cooperation’s evolution in such a manner, and they illuminate the underlying mechanisms by explaining a tradeoff between fixation probability and fixation time as well as the influence of time scale.

The revisions add to the richness of these findings. Among a number of sound comments, Reviewer #1 noted that the authors’ novel framework substitutes for rewiring and activity-driven models, respectively, which also provide a basis for understanding how network phenomena change when only a subset of edges are made to persist. In response to this comment, the authors repeated their investigation in, respectively, a rewiring model and an activity-driven model, finding that their key conclusion concerning the effect of heterogeneity remained robust across the two alternative modeling paradigms. In a similar vein, Reviewer #1 noted that the original version of the manuscript solely focused on the donor-recipient variant of the prisoner’s dilemma, thus limiting the generality of the findings; in response, the authors have extended the work meaningfully to consider the snow-drift game. Alongside these substantial changes that added to the robustness of their findings, the authors also implemented a range of suggestions from Reviewers #1 and #2, which varied from correcting notational mishaps to elaborating on points in the discussion section. Instead of enumerating all of those changes here, I simply would attest that the authors judiciously responded to all of the reviewers’ comments listed in their response letter.

Thank you for your effort on carefully reading and assessing our manuscript. We are very glad to hear your positive assessment of our manuscript and revisions.

The only suggestion that I believe continues to warrant some attention concerns the writing in the manuscript. Reviewer #1 raised this concern and I believe it still holds. The paper contains typos (e.g., the authors use the word “consisting” on page 7 when intending to use the word “consistent”) and the writing at times defies grammatical conventions in ways that might impede reader comprehension of the valuable science that the manuscript reports. For instance, the second sentence of the abstract states “While previous findings suggest that structural heterogeneity in static networks hinders cooperation, real-world interactions in most natural and social systems are

dynamic and best represented as temporal networks"; please note that this excerpt is an example of "faulty subordination" in the sense that the subordinate clause ("While … cooperation,") does not align with the main clause which focuses on real-world interaction whereas the subordinate clause focuses on findings. Such issues occur throughout the paper and might impede reader comprehension. Addressing these minor matters would ensure that the quality science at the heart of the paper is not clouded by correctable writing issues.

Thanks a lot for your constructive comments. We have checked through the manuscript and corrected all typos.

But, again, those are very minor matters that should not prevent publication. The paper presents a new modeling framework that will prove to be influential in the study of temporal networks and social evolution, and it uses this framework in an investigation that yields an important finding that overturns the field's understanding of how network heterogeneity affects cooperation.

We again thank you for your valuable comments and suggestions. Addressing them has considerably improved the quality of our manuscript.
